# Emerging trans-Eurasian heatwave-drought train in a warming climate

**DOI:** 10.1126/sciadv.adr7320

**Published:** 2025-05-02

**Authors:** Jee-Hoon Jeong, Min-Seok Kim, Jin-Ho Yoon, Hyungjun Kim, Shih-Yu Simon Wang, Sung-Ho Woo, Hans W. Linderholm

**Affiliations:** ^1^ Department of Environment and Energy, Sejong University, Seoul, South Korea.; ^2^ Department of Oceanography, Chonnam National University, Gwangju, South Korea.; ^3^ School of Earth Sciences and Environmental Engineering, Gwangju Institute of Science and Technology, Gwangju, South Korea.; ^4^ Moon Soul Graduate School of Future Strategy, Korea Advanced Institute of Science and Technology, Daejeon, South Korea.; ^5^ Institute of Industrial Science, University of Tokyo, Tokyo, Japan.; ^6^ Department of Agronomy, Kasetsart University, Bangkok, Thailand.; ^7^ Department of Earth Sciences, University of Gothenburg, Gothenburg, Sweden.

## Abstract

Since the late 20th century, an emerging atmospheric teleconnection pattern, the trans-Eurasian heatwave-drought train, has intensified remarkably during summer, correlating with a surge in concurrent heatwave-drought events from Eastern Europe to East Asia. Tree-ring proxies, spanning three centuries, reveal that the recent intensity of this pattern is unprecedented in the historical records. In contrast, the circumglobal teleconnection, which historically dominated the continental-scale Eurasian heatwave occurrences, has shown no discernible trend amid global warming. Consequently, this emerging pattern signifies a radical shift in Eurasian heatwave-drought climatologies. The mechanism involves Rossby wave propagation linked to warming sea surface temperatures in the Northwestern Atlantic and enhanced Sahel precipitation, both amplified recently by overlapping effects of anthropogenic warming and natural variability. Land-atmosphere interactions driven by soil moisture deficits further intensified the pattern regionally. Climate models predict that anthropogenic forcings will continue to strengthen the pattern throughout this century.

## Teaser

An emerging atmospheric teleconnection pattern drives a recent spike in trans-Eurasian heatwave-drought compounds.

## INTRODUCTION

In recent years, Eurasia has seen a surge in so-called heatwave-drought compounds, where intense heatwaves are accompanied by droughts (*1*, *2*). These events, which have far-reaching impacts on agriculture, water resources, and ecosystems, have become a growing concern. Studies widely attribute this phenomenon to global warming (*3*–*6*), highlighting the urgent need to diagnose and predict which regions are vulnerable to these unprecedented extremes to prepare for the devastating impacts of climate change in the future.

Historically, heatwave-drought compounds have been predominantly observed in so-called hotspot regions such as western North America, Australia, and western Europe (*7*, *8*). In the past few decades, their frequency has increased sharply across Eurasia, particularly in inland East Asia and Central Asia (*9*–*11*). This emerging trend raises an important question: Why are these extremes increasingly concentrated in specific regions? Is this pattern merely a reflection of local climate features, or does global warming create specific atmospheric conditions that favor such climate extremes?

Previous studies suggest that heatwave-drought compounds are driven by persistent high-pressure systems, which induce extreme heat and enhanced evapotranspiration, further amplified by local land-atmosphere interactions (*12*–*14*). These processes are closely linked to large-scale atmospheric circulation patterns, such as weakened jet streams and atmospheric blocking (*9*, *15*–*17*). Thus, if global warming strengthens certain atmospheric circulation patterns, then it could lead to such a systematic change in heatwave-drought compounds.

This study proposes an intensifying atmospheric circulation pattern that is driving a spike in the co-occurrence of heatwaves and droughts across the Eurasian continent and fundamentally changing the geography of their occurrence. By analyzing observational data including tree-ring proxies and climate model simulations, we demonstrate the relationship between the emerging atmospheric circulation pattern, increasing heatwave-drought compounds, and anthropogenic warming.

## RESULTS

### Robust trans-Eurasian heatwave-drought trends in recent decades

**Fig. 1.**
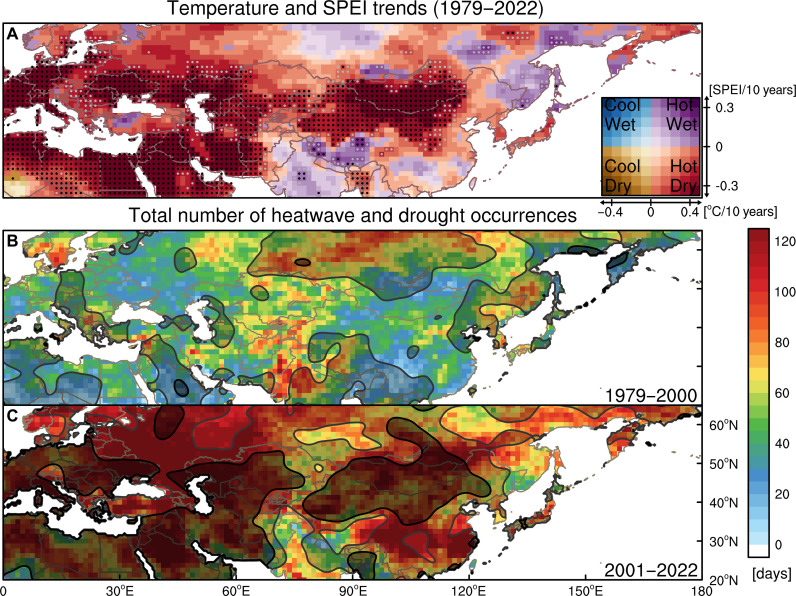
Warming-drying trends in Eurasia and marked changes in heatwave-drought occurrence pattern in recent decades. (**A**) Linear trends of July to August (JA) mean SAT (surface air temperature) and SPEI6 (SPEI over six-month scale) in 1979–2022. Gray dots mark the grids where both SAT and SPEI6 trends are statistically significant at the *P* < 0.10 level. Black dots mark the grids where have statistically significant trends after controlling for the false discovery rate (α_FDR_ < 0.10) (see the Materials and Methods for the detailed method). SAT trends (degrees Celsius per decade) are shaded at 0.1 intervals from −0.4 to 0.4 along the *x* axis of the inserted color matrix in the lower-right corner, and SPEI6 trends (per decade) are shaded at 0.075 intervals from −0.3 to 0.3 along the *y* axis of the same color matrix. The value of 0.4 is approximately the sum of the mean and 0.5 SDs (σ) of the SAT trends in absolute value over all grid points in Eurasia, while the value of 0.3 is that for SPEI6 trends. (**B** and **C**) Total number of JA heatwave (days, shading) and drought (months, contour) occurrences in 1979–2000 (B) and 2001–2022 (C). Gray and black contour lines represent the regions with over 8 and 14 drought occurrences, which are the average frequencies over Eurasia in 1979–2000 and 2001–2022, respectively.

Since around the 1980s, the summertime warming trend in Eurasia has been enormously strong (*18*, *19*). In recent decades (1979–2022), collective trends in summer (July to August) temperatures and cumulative Standardized Precipitation Evapotranspiration Index [SPEI; (*20*)] over Eurasia (Fig. 1A; see caption) show a telling pattern that outlines a hotter and drier Eastern Europe, Black Sea Rim, Iranian Plateau, and Mongolia-North China region, as well as Ukraine. The warming trend is relatively weaker in Central Asia and higher latitudes.

Further examination of the changes in extremes, heatwave and drought events (see the Materials and Methods for the definition) in the decades before and after the year 2000 reveals a marked geographical shift alongside the pronounced increase in the occurrence frequency of such events in Eurasia (Fig. 1, B and C). In the late 20th century (1979–2000), heatwaves were concentrated in Central Siberia, Northwest India and Central Asia, and China and the Far East, while droughts were mostly distributed in Central Siberia and Northwest India. Notably, in recent two decades (2001–2022), this pattern has almost reversed, with the greatest number of heatwaves now occurring in regions where they rarely occurred previously, e.g., European Russia and Southern Europe, Inner East Asia, and Southern China, where the number of heatwaves has nearly quadrupled. For droughts, the most severe ones in recent decades have occurred in eastern and southern Europe, the Middle East and northern Africa, the Caucasus, Mongolia, and northern China, coinciding with the increased occurrence of heatwaves. However, in the previous period, there was not a strong spatial connection between heatwave and drought hotspots. The intensity of heatwaves and droughts also changed in much the same pattern as their occurrence frequency [fig. S1 and (*16*)]. Consequently, the synchronization of the two extremes has subsequently become pronounced. Previous studies suggested that this feature could be a result of enhanced land-atmosphere coupling triggered by drying soils (*17*, *21*–*23*), and the trend toward a hotter-drier inner East Asia, around Mongolia, could be indicative of an irreversible regional climate change (*9*). Nevertheless, the mechanism underlying this abrupt but systematic change across the Eurasian region remains largely unexplained.

### Atmospheric teleconnection behind compound heatwaves droughts

**Fig. 2.**
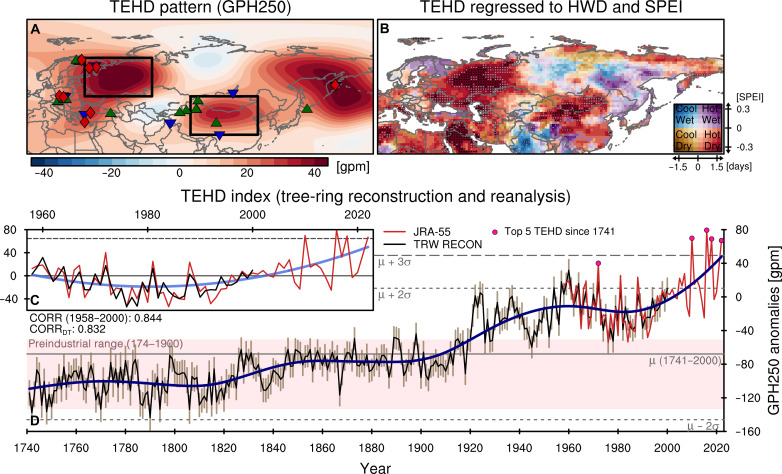
Trans-Eurasian heatwave-drought train (TEHD) pattern and variability. (**A**) The TEHD pattern represented by the leading empirical orthogonal function mode for JA mean GPH250 (geopotential height at 250 hPa) anomalies over Eurasia (0° to 180°E, 20° to 90°N) in 1979–2022. Black frames denote two TEHD cores, European Russia (30° to 65°E, 50° to 70°N) and Northeast Asia (85° to 120°E, 30° to 50°N). Green triangles, blue reversed-triangles, and red diamonds mark the sites where tree-ring data were used to reconstruct the TEHD variability on above-interannual timescale, interannual timescale, and both timescales, respectively. (**B**) Regression coefficients of JA total heatwave days (HWD) and mean SPEI6 anomalies with respect to the *z* score standardized TEHD index (1979–2022). Gray dots denote the grids with significant coefficients (*P* < 0.10) for both HWD and SPEI6. Black dots denote the grids with significant coefficients after controlling for the FDR (α_FDR_ < 0.10). (**C** and **D**) Temporal evolution of the TEHD index from JRA-55 (red; 1958–2022) and the tree-ring reconstruction (black; 1741–2000). In (C), blue line represents the nonlinear trend, represented by the residual component decomposed by the Ensemble Empirical Mode Decomposition (EEMD). Gray solid and dotted lines represent the mean and 2σ of the observation (1979–2022), respectively. In (D), navy line represents the low-frequency (≥50 years) variation of the TEHD index (1741–1957: reconstruction, 1958–2022: JRA-55), defined as the sum of the fifth to the last intrinsic mode functions (IMFs) and the residual by the EEMD. Gray solid, dotted, and dashed lines represent the mean, 2σ, and 3σ of the reconstruction (1741–2000), respectively. Pink shading represents 2σ range from the mean of the reconstruction for the preindustrial period (1741–1900). Pink dots denote the five strongest TEHD years. Beige bar indicates the reconstruction uncertainty defined as ±1 RMSE (root mean square error). gpm, geopotential meters.

The emergence of concurrent heatwaves and droughts across Eurasia is not merely a coincidence but rather an intertwined pattern of large-scale atmospheric circulation and land surface changes (*24*–*26*). Figure 2A delineates the salient feature of upper-tropospheric atmospheric circulation changes (geopotential height at 250 hPa) across the Eurasian continent in recent decades: a conspicuous wave train pattern emanating from Eastern Europe, traversing Central Asia, and extending to the Kamchatka Peninsula. The strongest center of this wave train lies in the European Russia region, with the highest pressure anomaly adjacent to low-pressure anomalies in the vicinity of Uzbekistan-Turkmenistan and the northern areas of Siberia. Further downstream, pronounced high-pressure anomaly centers appear in Mongolia and Northern China, coinciding with the increased heatwaves and droughts. We defined this prominent atmospheric circulation pattern as the trans-Eurasian heatwave-drought train (TEHD) and investigated its long-term variation by estimating its intensity from the upper-level geopotential height of two distinct high-pressure centers (see details in the Materials and Methods). In Fig. 2B [more in fig. S2 (A to D)], the regression coefficients of heatwave days and SPEI anomalies with respect to the TEHD index during 1979–2022 successfully record the trending patterns of drought and heatwave as shown in Fig. 1.

Throughout the reanalysis data period (post-1958), the TEHD pattern seems to fluctuate with multidecadal cycles (Fig. 2C). A low-frequency variability is observed where the pattern weakens from the 1960s through the early 1990s and then strengthens after the 1990s in a seemingly permanent way, reaching historically high values in the 2000s. Expectedly, this wave train is also evident in the interannual and subseasonal timescales (fig. S3). During the years when the TEHD index exceeded 2 SDs (σ) of its historical variability, such as 2010, 2016, 2018, and 2022, record-breaking heatwave-drought compounds in Eurasia were found (*21*, *22*, *27*–*33*). In particular, the famous 2010 Russian heatwave, which caused 55,000 deaths along with drought and wildfires (*17*, *34*–*36*), and the 2022 heatwave that hit East Asia causing flash-droughts (*37*), are typical examples of events that occurred in conjunction with strong TEHD patterns. The high-pressure anomalies in the two central TEHD regions are correlated on a subseasonal timescale; when a high-pressure appears in European Russia, a high-pressure emerges in Inner East Asia about a week later (fig. S3D). This correlation has become more pronounced and lasted longer in recent decades. Given the prominence of the TEHD pattern and its observed association with amplified heatwaves and droughts, one has to ask: Does this pattern represent a preferred mode in the Eurasian continent under global warming? To what extent does the TEHD intensification compare with past variability?

To answer these questions, we produced the tree-ring based reconstruction of TEHD variability to examine how it evolves over an extended timescale. Traditionally, tree-ring data have predominantly served to reconstruct temperature and hydroclimate variables, which directly influence tree growth (*7*, *9*, *19*). However, given that the atmospheric circulation exerts a multifaceted influence on plant growth environments, affecting climate factors such as temperature, solar radiation, and precipitation, tree-rings are well-positioned to capture such influences effectively (*38*, *39*). More specifically, the subsidence from the anomalous ridge over the TEHD core regions, when the TEHD is active, suppresses convection and precipitation, elevates solar radiation and temperature, and reduces humidity, affecting tree growth synthetically (*12*, *40*–*42*). This suggests an idea that collective records of tree growth, in turn, can be used to infer the strength of TEHD patterns. Recent studies have successfully used tree-ring data to restore long-term variations in large-scale atmospheric circulation patterns, such as the El Niño–Southern Oscillation, the Pacific Decadal Oscillation, the Arctic Oscillation, the North Atlantic Oscillation, the Atlantic Multidecadal Oscillation (AMO), the Pacific North America, the Northern Hemisphere jet stream, and the Siberian High (*43*–*52*).

Here, we extracted tree-ring chronologies from 33 sites (Fig. 2A and table S1) across the Eurasian continent deemed sensitive to TEHD variability. The selected tree-ring data are distributed across the TEHD’s core and nearby regions, allowing the extraction of large-scale, broad features of the TEHD rather than specific local features. In alignment with the methodology used by Zhang *et al.* (*9*), we segregated the predictand (our designated reconstruction target, the TEHD index) and the predictors (tree-ring proxies) into their respective long-term and short-term variabilities. We then reconstructed them separately and reassembled them to reconstruct the TEHD index (see the Materials and Methods for more details). On the basis of this strategy, we produced a robust reconstruction of TEHD variability over the past 282 years (1741–2022) (fig. S4). Figure 2D illustrates the reconstructed TEHD index, which accounts for 71.19% (*r* = 0.844) of the observed variance spanning the years 1958–2000. In addition, the reconstructed multidecadal variation of the TEHD in early 20th century agrees well with that from the extended reanalysis, the NOAA 20CR (National Oceanic and Atmospheric Administration’s 20th Century Reanalysis) (fig. S5, A and B).

While the reconstructed TEHD index exhibits a positive trend across its entire timespan, its trajectory varies considerably depending on both the temporal scale and specific epochs (Fig. 2D). In the early part of the record, from the 1740s to the 1810s, coinciding with the Little Ice Age, the TEHD remained at historically low levels but with noticeable decadal variability. It rose in the 1820s and showed a sharp dip during the Dalton Minimum in the 1840s but continued to rise through the 1850s. This implies that the TEHD is an essential pattern in the climate system, which it can arise from natural variability. It then remained stable and less variable until the 1910s when it began to rise rapidly. After a brief dip in the early 1940s, but the upward trend continued through the 1960s. It then dipped until the 1980s, after which it began to rise again, reaching historic levels (exceeding +3σ) repeatedly in recent years. Nearly every year, the TEHD has exhibited intensities surpassing 2σ of the entire reconstruction range (1741–2000) since the 2000s. Four of the top five most pronounced TEHD events occurred during the post-2010 period. Similarly, the years 2010, 2016, 2018, and 2022 coincided with unprecedented heatwaves and droughts in Eurasia (*21*, *22*, *27*–*33*). This analysis underscores the recent amplification of the TEHD, transcending natural variability.

### Trans-Eurasian teleconnection pattern prevailing with global warming

**Fig. 3.**
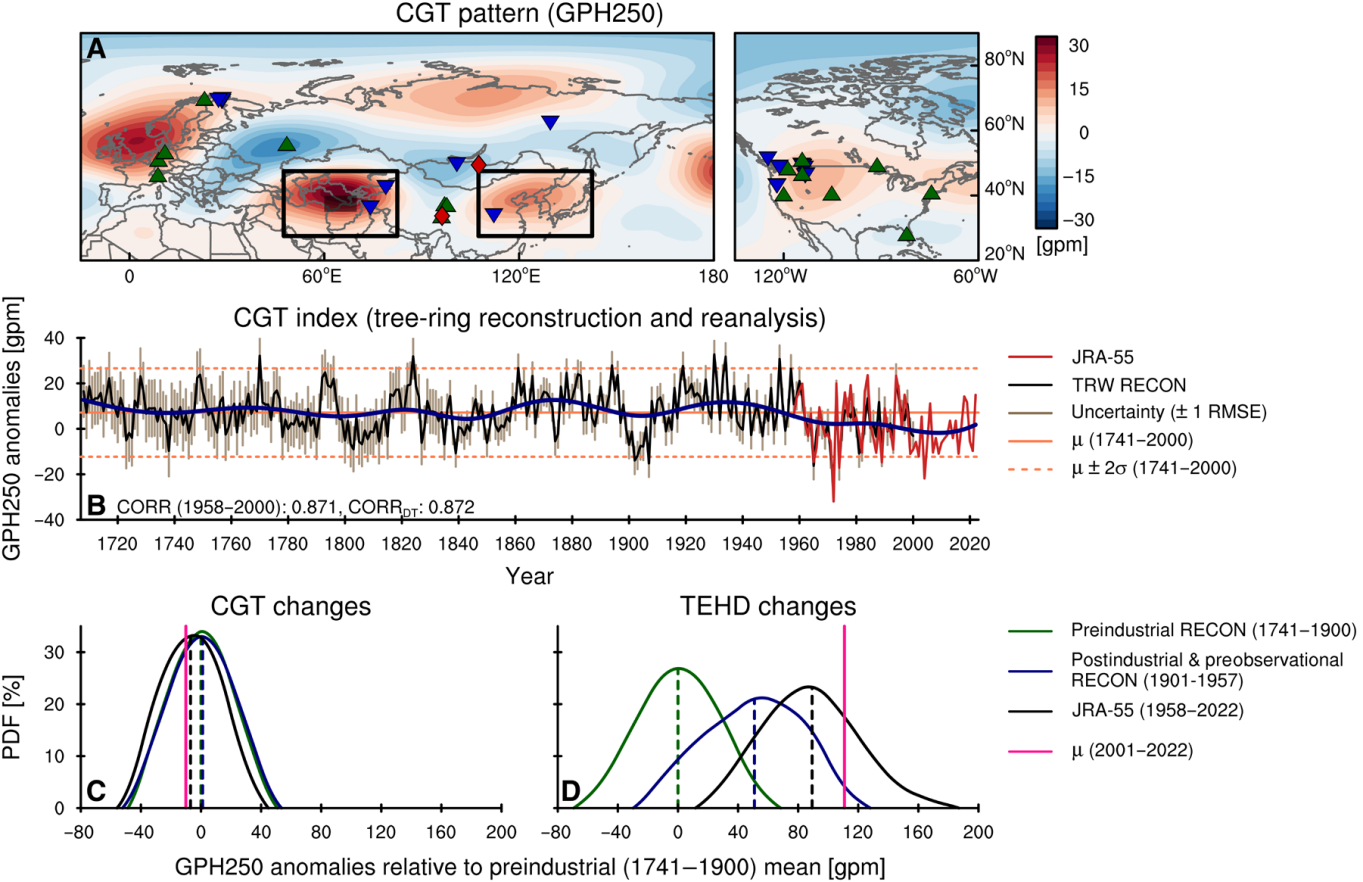
Comparison with long-term changes in the circumglobal teleconnection (CGT). (**A**) The CGT pattern represented as regression coefficients of JA GPH250 anomalies with respect to the *z* score standardized CGT index in 1979–2022. Green triangles, blue reversed-triangles, and red diamonds are same as described in Fig. 2 but for the tree-ring data used in the CGT index reconstruction. (**B**) Temporal evolution of the CGT index from JRA-55 (red line) in 1958–2022 and the tree-ring reconstruction (black line) in 1708–2000. Orange solid and dotted lines represent the mean and 2σ of the reconstructed index for 1741–2000, respectively. Navy line represents the low frequency (≥50 years) of the CGT index (1708–1957: reconstruction, 1958–2022: JRA-55). Beige bar indicates the uncertainty of the reconstruction (±1 RMSE). (**C** and **D**) The probability distribution function of the CGT index (C) and the trans-Eurasian heatwave-drought train (TEHD) index (D) for the periods 1741–1900, 1901–1957, and 1958–2022. Vertical dashed lines represent the mean of each period. Pink solid line represents the mean of each index for 2001–2022.

Previous studies have identified another prominent pattern of atmospheric circulation variability, namely the well-known circumglobal teleconnection (CGT) as the primary driver behind heatwaves across the Eurasian continent (*31*, *53*). The CGT is initiated by upper-tropospheric circulation disturbances originating near the entrance of the south Asian jet, in proximity to Northwestern India (*53*–*55*). With the trapped Rossby wave energy within the jet stream, the CGT traverses the Northern Hemisphere’s subtropical regions, manifesting as alternating high- and low-pressure wave trains (Fig. 3A). When the CGT is active, it facilitates the formation of anticyclonic circulations in various Eurasian regions, including Northern Europe, West Asia, East Asia, and northwestern North America. These regions, marked by positive centers of action, often experience heatwave (fig. S2H). During the late 20th century, the CGT was vigorous for many years (fig. S2J) resulting in concentrated heatwave occurrences underneath the CGT core regions (Fig. 1B). Particularly, in 1984 and 1994, when the CGT intensity peaked at historical levels, record-breaking number of heatwaves were registered in those core regions (*35*, *56*–*60*). However, in the early 21st century, the CGT has weakened, in contrast to the strengthening TEHD (fig. S2, E and J).

Compared to the TEHD, the CGT’s wave trains have their centers of action concentrated in the Eurasian continent and the North American subtropics (c.f. Figs. 2A and 3A). Nevertheless, the low-high pattern observed over western Russia and Central Asia bears some resemblance to the TEHD pattern, albeit with inverted signs. This similarity is likely because these regions lie downstream of the jet exit (*61*), resulting in a comparable magnitude of response to the wave source. However, outside of Central Asia, where the two patterns appear to overlap, the two patterns become distinct, and further analysis of upper-level pressure data across the Northern Hemisphere (fig. S6) reveals that the TEHD and CGT are more clearly distinguishable as independent modes. Furthermore, the perturbations linked to the TEHD, which have recently intensified and become more extreme, seem to exert more severe effects; their interaction with hydroclimatic variables demonstrates a noticeable difference from the CGT. While TEHD has a strong coupling between hot and dry conditions (heatwaves and droughts) across its two high-pressure centers, this is not the case for the CGT (fig. S2, F to I). This suggests a difference in the duration of events, where the TEHD tends to last longer than the CGT, prompting hot conditions to persist longer and form drier conditions.

To compare the historical changes of the two modes, we also produced a reconstruction of past CGT’s variability from 1708 to the present, explaining 75.84% of the observed variance for the period 1958–2000 (Fig. 3B and fig. S7). The CGT reconstruction used 38 tree-ring chronologies from Eurasia and North America, none of which overlap with those used in the TEHD reconstruction. While the reconstructed CGT also exhibits multidecadal-to-centennial oscillations, it does not show a noticeable trend in recent decades. Whereas the TEHD showed a rapid strengthening from the 1980s to the present, the CGT has been showing rather a weak negative trend. However, this decline does not exceed its internal variability, as its variations in the past few decades fall within the range of variability over the past 300 years (Fig. 3, B and C). In recent decades, CGT has continued to induce heatwave trains in the Eurasian region, such as in 2003, 2006, 2015, and 2018 (*31*). However, no particularly strong CGT cases have been observed after the 2000s (Fig. 3B and fig. S2J). By comparison, the recent overwhelming enhancement of TEHD, which is unprecedented in a preindustrial context (Fig. 3D), has resulted in the emergence of Eurasia heatwave-drought trains, suggesting that it is a preferred mode of anthropogenic climate change.

### Underlying mechanisms

**Fig. 4.**
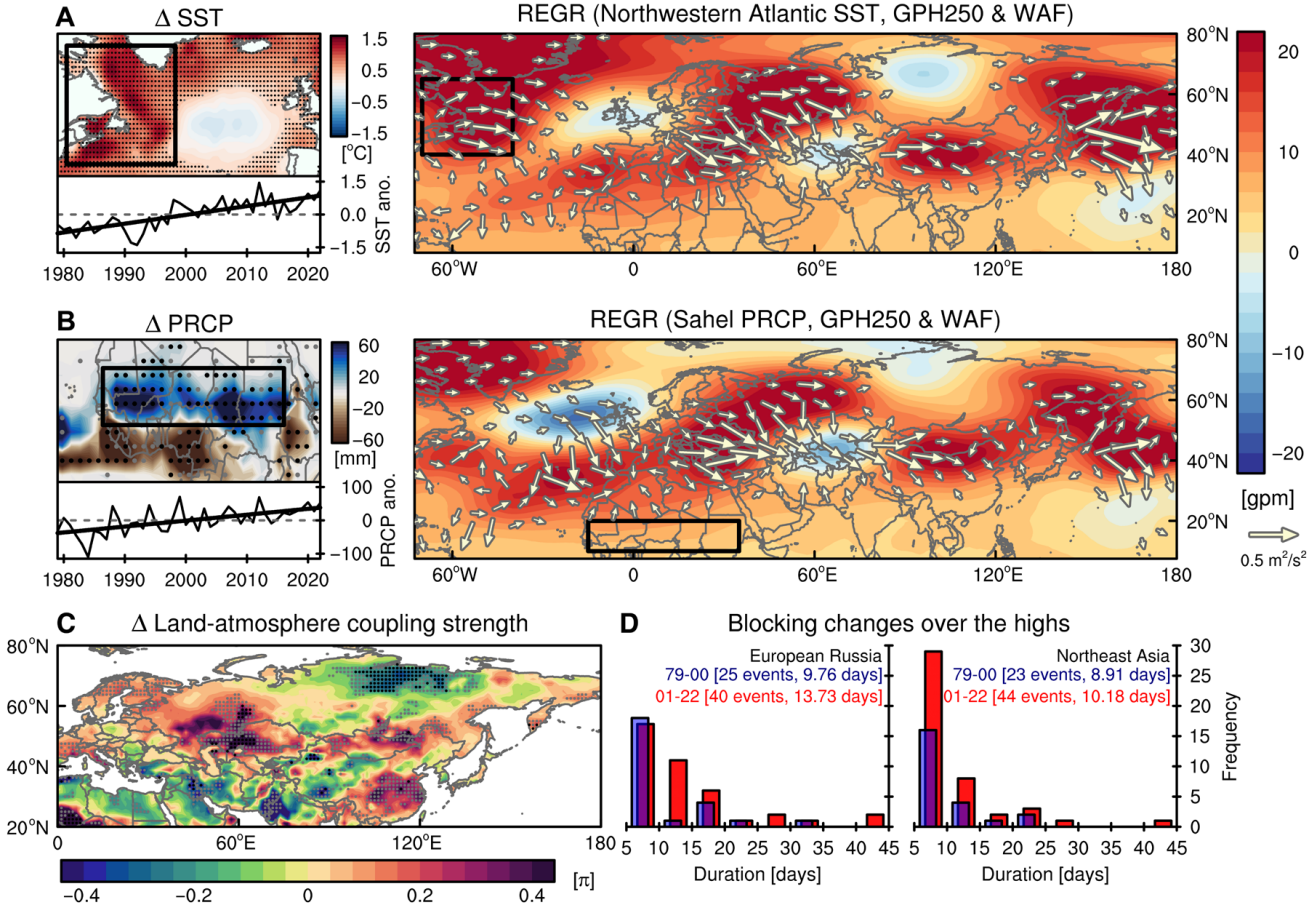
Underlying mechanisms behind trans-Eurasian heatwave-drought train (TEHD) strengthening. (**A**) Mean differences of JA mean sea surface temperatures (SSTs) from 1979–2000 to 2001–2022 (recent minus past) and JA SST anomalies averaged over the Northwestern Atlantic (40° to 70°W, 40° to 65°N) with its linear trend (black thick line) in 1979–2022 (left). Regression coefficients of JA mean GPH250 anomalies (shading) and WAF (vector; see the Materials and Methods for the calculation method) with respect to the *z* score standardized SST anomalies averaged over the Northwestern Atlantic in 1979–2022. (**B**) Same as (A) but for the total precipitation (PRCP) changes over the African Sahel (15°W to 35°E, 10° to 20°N). (**C**) Mean differences of JA mean land-atmosphere coupling strength from 1979–2000 to 2001–2022. In (A) to (C), gray dots denote the regions where mean differences are statistically significant at the *P* < 0.10 level. Black dots mark the grids where have statistically significant values after controlling for the false discovery rate (FDR) (α_FDR_ < 0.10). (**D**) Histogram of blocking-high duration for European Russia (left frame in Fig. 2A) and Northeast Asia (right frame in Fig. 2A) in 1979–2000 (blue bars) and 2001–2022 (red bars). The numbers in square brackets indicate the total number and mean duration of blocking occurrences in each region during each period. REGR, regression.

The recent intensification of TEHD appears to be a cumulative outcome of enhanced Rossby wave sources and amplified land-atmosphere coupling. One such source of Rossby wave energy is the strong sea surface temperature (SST) warming over the Northwestern Atlantic, where the SST anomaly is strongly associated with upper-level Rossby wave propagation, forming a pattern that closely resembles the TEHD pattern as shown by the geopotential height and wave activity flux (WAF) regressions [Fig. 4A, (*62*, *63*)]. The North Atlantic has experienced strong warming trend since the mid-1990s, due to the combined effects of global warming and the transition from a negative to a positive phase of the AMO, resulting in record warmth in recent years (Fig. 4A and figs. S5C and S8) (*64*–*66*). Thus, this oceanic warming could contribute to the TEHD intensification.

Another corresponding source is the increasing precipitation in the African Sahel region over the same timeframe, marked by an enhancement in monsoonal convection, a feature associated with the AMO turnover (*65*, *67*) and human-induced climatic shifts (*68*). The anomalous heating related to Sahel precipitation drives a Rossby wave propagation that is also akin to the TEHD pattern [Fig. 4B and (*69*)]. The observational record available since the early 20th century does not show an overall increase in Sahel precipitation due to global warming (fig. S5D), with a more pronounced multidecadal oscillation associated with the AMO. Still, the pronounced increase in Sahel precipitation since the 1980s is likely to have contributed to the recent intensification of the TEHD pattern.

Both wave patterns manifest as a local high-pressure anomaly in regions surrounding the Black Sea-Caspian Sea and elevated regions in Mongolia. The rapid drying trends experienced in these areas (Fig. 1A) also enhance the land-atmosphere coupling where drier soil further heats the air above it and intensifies high-pressure and vice versa (Fig. 4C and fig. S9) (*9*, *23*). Consequently, atmospheric blocking events in these two heatwave-drought hotspots, defined as daily 250-hPa geopotential height exceeds 1σ above the summer mean over 5 consecutive days (*70*), seem to occur more frequently and persist longer in the recent decades, therefore sustaining the TEHD pattern for extended durations (Fig. 4D). These effects, combined with the global geopotential height rise due to global warming (fig. S10), appear to have led to a record-breaking intensification of high-pressure systems over the TEHD regions in recent decades.

### Attribution and future projections by climate models

**Fig. 5.**
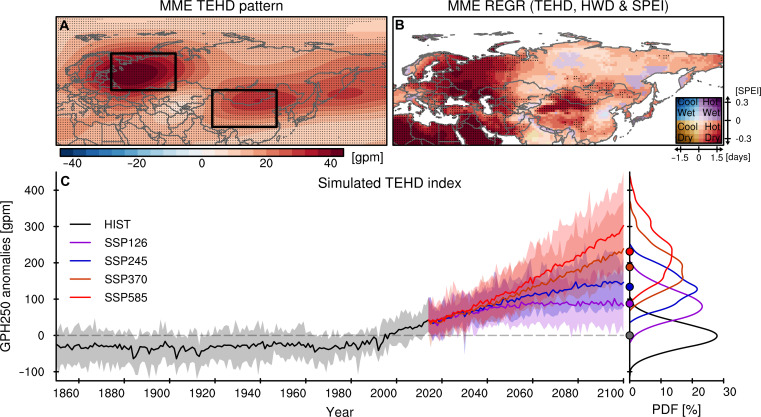
Trans-Eurasian heatwave-drought train (TEHD) simulated by climate models and future projections. (**A** and **B**) Multimodel ensemble mean (MME) regression coefficients of JA mean GPH250 (A) and total HWD and mean SPEI6 (B) anomalies with respect to *z* score standardized TEHD index in 1979–2014 from the 21 CMIP6 models. Dotted areas represent regions where 17 or more models (about 80% of the total number of models) show the same sign with the MME response. (**C**) MME TEHD index from CMIP6 historical simulations in 1850–2014 and future projections based on four SSP scenarios in 2015–2100. Shaded areas indicate the ensemble spread of the models. The figure attached to the right represents the probability distribution function of the TEHD index in 1979–2014 (historical simulations) and 2065–2100 (future projections with the four scenarios) from the model ensembles with their mean value (filled circles).

To attribute the historical TEHD strengthening and its potential future alterations, we examined the changes in TEHD patterns based on Coupled Model Intercomparison Project Phase 6 (CMIP6) simulations under a historical scenario and the SSP (Shared Socioeconomic Pathways) future scenarios (*71*). While the amplification of TEHD involves intricate interactions among the atmosphere, land, and ocean, which led to considerable disparities across models, most of the models participating in CMIP6 (fig. S11) closely mimic the spatial patterns of the real-world TEHD in their ensemble mean (Fig. 5, A and B). The pronounced intensification of TEHD patterns in the late 20th century is also apparent in the historical scenario, and models project strengthening in all four SSPs (Fig. 5C). The prevailing SST warming trend in the Northwestern Atlantic and the enhancement of monsoon rains in the African Sahel—factors that contribute to the intensification of TEHD—are also represented in the CMIP6 historical simulation (fig. S12). This does not include the Northwestern Atlantic warming effect linked to the multidecadal oscillation in the observations, but the anthropogenic warming effect is driving substantial levels of warming. Moreover, despite a single cessation of TEHD enhancement in the SSP126 scenario, all other scenarios project proportionally more intense global warming leading to stronger amplification of TEHD throughout the latter half of the 21st century (Fig. 5C), solidifying the effect of anthropogenic warming on such teleconnections. In contrast, natural-only historical CMIP6 simulations, without anthropogenic greenhouse gas and aerosol forcings [hist-nat simulations; (*72*)], are unable to reproduce the observed intensification of TEHD, indicating its anthropogenic origin. For the preindustrial CMIP simulations [piControl; (*71*)], the TEHD pattern is similarly found, although its amplitude is slightly smaller than in the historical and SSP simulations (fig. S13). Still, the strength of TEHD intensification seen in observations and historical simulations exceeds the natural variability in the piControl (fig. S14). This suggests that the TEHD is an inherent pattern that resides in the climate system but has been greatly intensified in response to anthropogenic forcings.

Regarding the feedback effect from land-atmosphere interaction, the CMIP6 historical simulation also simulates recent drying in the TEHD core region, which is projected to intensify in the future. Analyses of the land-atmosphere feedback effect using the Land Feedback Model Intercomparison Project [LFMIP-pdLC; (*73*)] simulations, an experiment that constrains soil moisture feedbacks (fig. S15), show that the feedbacks of high-pressure intensification and temperature increase due to soil drying in the TEHD core region are contributing to the intensification of TEHD, which supports observational evidence.

For the CGT, which showed a predominant natural variability without any notable trends in observational data and reconstructions, CMIP6 models project it to strengthen throughout the 21st century (fig. S16). Although our CMIP6 analysis shows that the CGT may strengthen, its magnitude is noticeably weaker than the TEHD. Therefore, the CMIP6 scenarios still suggest the TEHD pattern induced by global warming will remain the dominant mode, likely leading to an amplification of heatwave-drought compounding events in Eurasia during summer. These modeling outcomes further bolster the assertion that the recent growth of the TEHD and its association with the profound increases in Eurasian heatwaves and droughts is a consequence of human-induced climate alterations. Furthermore, the impact of the intensified TEHD on future heatwaves and droughts over the core and peripheral regions of the TEHD pattern across Eurasia is projected to be stronger under higher emission scenarios (fig. S17), suggesting that without substantial mitigation efforts, the TEHD-driven compounding of heatwaves and droughts will likely intensify further in Eurasia during the late 21st century.

## DISCUSSION

Tree-ring and observational evidence reveals a fundamental shift in the geography of summer heatwave-drought events over the Eurasian continent in recent decades. This is driven by an emerging large-scale atmospheric circulation pattern, the TEHD, which has spiked as an unprecedented strength. The emergence of TEHD is attributed to enhanced Rossby wave activity, triggered by warming in the Northwestern Atlantic and increased precipitation in the Sahel, resulting from the combined effect of global warming and partly by the AMO turnover. The land-atmosphere interaction has further fueled the amplification of the TEHD pattern. Climate models predict that TEHD will continue to intensify if global warming continues.

However, it is important to recognize the substantial uncertainties in the observational data and modeling results used to support these conclusions. Aside from the uncertainties in the tree-ring reconstructions, the observational record of multidecadal variability in Northwest Atlantic SST and Sahel precipitation is relatively short and limited in reliability. Furthermore, the impact of this natural variability is inherently difficult to diagnose in CMIP6 modeling results (*74*).

Despite these limitations, a range of evidence presented in this study strongly indicate that anthropogenic warming is fostering a “preferable pattern” of atmospheric circulation—TEHD—which amplifies the risks of heatwave-drought compounding extremes across Eurasia. The consequences of this shift are profound, with significant implications for wildfires, agriculture, food security, water resources, and ecosystems. Given that future simulations predict continued intensification of these changes, urgent mitigation efforts are needed to address the escalating risks.

## MATERIALS AND METHODS

### Definition of heatwave and drought

Heatwaves are identified when there are at least 3 consecutive days with daily maximum near-surface air temperatures (*T*_max_) above the 90th percentile, calculated for each calendar day using a 15-day moving window from 1979 to 2022 (*75*). This approach considers temporal dependence and ensures a sufficient sample size for accurate percentiles. Daily thresholds for heatwaves are calculated for each grid box. Two heatwave characteristics were investigated. First, summer heatwave frequency is the total number of heatwave days. Second, cumulative heatwave intensity (cumulative heat) is the sum of the *T*_max_ anomalies compared to the calendar-day 90th percentile during heatwave days (*75*). In this study, “summer” is defined as the period from July to August, which is typically when Eurasia experiences the most heatwaves. Heatwave characteristics were analyzed using daily *T*_max_ data from the JRA-55 (*76*), covering the period from 1958 to 2022.

Drought events in this study are detected using the monthly SPEI6 (6 months SPEI), from the global SPEI database (SPEIbase) version 2.9 (*20*) covering the period from 1901 to 2022. SPEI is effective for detecting cumulated dry conditions as it accounts for both precipitation and evapotranspiration. A use of SPEI1 and SPEI3 (1 month and 3 months SPEI), which represent the cumulative effect over a shorter period, more markedly reveal the changes in the heatwave-drought compounds, but in this study, SPEI6 was used to focus on extreme seasonal droughts. Smaller negative SPEI values indicate stronger dryness, and for this study, a drought is identified when SPEI6 is less than or equal to −1. Two drought characteristics are calculated: frequency, which is the total number of drought months and cumulative intensity, or cumulative dryness, which is the sum of SPEI6 anomalies compared to the −1 threshold during the drought months.

### The TEHD and CGT indices based on atmospheric circulation

The TEHD pattern is defined as the leading mode of the empirical orthogonal function for July to August (JA) mean geopotential height anomalies at 250 hPa (GPH250) over Eurasia (0° to 180°E, 20° to 90°N) during 1979–2022. It explains 27.78% the total JA mean GPH250 variations over Eurasia. While this study primarily uses the JRA-55 dataset, other reanalysis datasets (table S2) (*77*–*81*) have similarly identified the TEHD pattern as the dominant summer upper-level atmospheric circulation pattern over Eurasia. The TEHD index is calculated as the mean of two area-averaged JA mean GPH250 anomalies over European Russia (30° to 65°E, 50° to 70°N) and Northeast Asia (85° to 120°E, 30° to 50°N), which are the core regions of the TEHD pattern (indicated by black frames in Fig. 2). Direct averaging of two time series can lead to biases due to varying GPH250 variances across these regions. To mitigate this, each time series was *z* score standardized over the period 1979–2022 before averaging. The averaged, standardized index was then adjusted by multiplying it with the mean value of regression coefficients of this index against GPH250 anomalies over the two core regions. This method enables the index to intuitively represent variance-corrected mean changes of GPH250 anomalies in the unit of geopotential meters. Furthermore, instead of using a principal component (PC) time series to quantify the TEHD intensity, this approach enables a concentration on the particular regions where the TEHD exerts the strongest influence on climate variability and extremes and is more effective at capturing the local-scale processes and feedbacks that contribute to the maintenance and amplification of the TEHD pattern. The defined TEHD index correlates well (*r* = 0.904 for 1958–2022) with the PC time series (PC1) of the TEHD pattern. In field regression and composite analyses to examine recent climatic trends and relationships with the TEHD, we used the Benjamini-Hochberg false discovery rate (FDR) correction for *P* values to minimize the number of false positives from multiple testing (*82*–*84*), following the methodology used in (*48*).

The CGT index is conventionally defined as the areal-averaged JA mean GPH250 anomalies over the CGT core region (indicated by the black filled box in Fig. 3A; 60° to 70°E, 35° to 40°N) as suggested by Ding and Wang (*53*). However, this is not directly comparable to the TEHD index above. To address this, a correction was applied by multiplying the *z* score standardized CGT index by the mean value of regression coefficients of this index with GPH250 anomalies over South Asia (47.5° to 82.5°E, 27.5° to 47.5°N) and East Asia (107.5° to 142.5°E, 27.5° to 47.5°N), which are considered the core regions of the CGT pattern over Eurasia (indicated by black frames in Fig. 3).

### Reconstruction of TEHD and CGT index

To reconstruct the long-term variability of the TEHD and CGT indices, tree-ring width (TRW) measurements from the International Tree-Ring Data Bank (ITRDB) were used. A total of 5239 TRW measurements from the Northern Hemisphere were initially downloaded. To eliminate the nonclimatic (biological) growth trends in trees, the data were standardized using the software program CRUST [Climatic Research Unit Standardisation of Tree-ring data; (*85*, *86*)]. Particularly, the Signal-Free Regional Chronology Standardization (fit by an age-dependent spline) method, detailed by Melvin and Briffa (*85*), was applied to preserve as much long-term climatic variability as possible while minimizing artifact trend distortions (known as end-effects). During the standardization process, because of formatting issues leading to program errors, some records were truncated. Consequently, 5063 TRW chronologies were successfully created, with 2087 from Eurasia and 2976 from North America. Of these, only the chronologies covering at least the period from 1850 to 2000 and having an Expressed Population Signal value of 0.85 or higher were retained (*87*). 1850 notes the start year of the CMIP6 historical simulations; thus, it was set up to get longer reconstructions than the climate model simulations. This resulted in 354 Eurasian and 640 North American TRW chronologies. For the TEHD index reconstruction, only Eurasian TRW chronologies were used, and for the CGT index reconstruction, both Eurasian and North American TRW chronologies were used.

Although the TEHD is a large-scale pattern covering the entire Eurasian continent, its impacts are heterogeneous and may be marginal in some areas. Furthermore, tree growth is affected by various climatic and nonclimatic factors, which means that some Eurasian TRW chronologies may not display any TEHD-related signals. Therefore, the TRW chronologies were screened by assessing their linear relationship with TEHD variability. The selected TRW chronologies had a statistically significant (*P* < 0.10) correlation with the TEHD index. In addition to this, to account for TEHD-related SAT (surface air temperature) change signals retained in the TRW chronologies, we examined two additional conditions. First, we used only the TRW chronologies that had a positive correlation with the local SAT variability (SAT anomalies averaged over the nine closest grid points surrounding the TRW data sampling site). Negative associations can often be indirectly caused by heat-induced water stress. We then tested whether the product of the correlation between the TEHD index and local SAT and the correlation between the TEHD index and TRW chronology was positive. For instance, if a specific region corresponded to a negative temperature anomaly in the TEHD pattern and tree growth over the region was positively associated with local SAT, then the TEHD index and the regional TRW chronology would have a negative correlation. The sign of the product of the two correlations being positive can guarantee their physical linkage, even at a minimal level. These approaches effectively selected TRW chronologies that contained SAT signals related to the TEHD pattern. In addition, we examined whether the TRW chronologies retained signals of TEHD-related SPEI changes in the same manner because the TEHD can also induce hydroclimate impacts on tree growth.

In recent dendrochronological studies, reconstruction models have been effectively established by separating high- and low-frequency variabilities in both the predictors (proxies) and the predictands (*9*, *19*, *88*). A key method in this process is the Ensemble Empirical Mode Decomposition (EEMD), detailed by Wu and Huang (*89*), which efficiently decomposes time series into a collection of intrinsic mode functions (IMFs) and a residual nonlinear trend. These IMFs represent oscillatory components at various frequency scales. Following the approach used by Zhang *et al.* (*9*), this study applied the EEMD method to analyze the long-term variabilities of the TEHD index and TRW chronologies. We decomposed these variabilities into interannual (defined here as 2 to 4 years and represented by the first IMF) and above-interannual (over 4 years, represented by the sum of the remaining IMFs and the residual) components. Subsequently, we constructed two separate reconstructions for these timescales.

Likewise, some of the selected TRW chronologies may be more sensitive to the long-term variability of the TEHD, while others may be more sensitive to its short-term variability. It should be noted that the preliminary screening work was based on the correlation between the original TEHD index and TRW chronology, without any filtering. Thus, to build the best performing reconstruction models for each decomposed timescale, it was tested whether iteratively removing the TRW chronology which had the lowest correlation with the TEHD index on the decomposed timescale, one by one, could yield substantial improvement. This ensures the use of the TRW chronologies with high fidelity, which contain meaningful signals of the TEHD in both the original and filtered variabilities. The selected TRW chronologies also had positive correlation with the local SAT/SPEI on the decomposed timescale, and the product of the correlation between the TEHD index and the local SAT/SPEI and the correlation between the TEHD index and the TRW chronology was positive on the decomposed timescale. Consequently, 18 TRW chronologies were used for the TEHD index reconstruction on an interannual timescale, while 23 TRW chronologies were used for above-interannual timescales. Eight TRW chronologies were used in both timescales (table S1). This combination resulted in the best regression models with the highest correlation between observed and reconstructed indices. But other combinations with fewer or more TRW chronologies also yielded similar variabilities, confirming the robustness of the reconstructions. For the CGT index reconstruction, following the same approach as the TEHD index, 22 TRW chronologies were selected to build reconstruction model on interannual timescale, and 18 TRW chronologies were selected for the above-interannual timescale, with 2 TRW chronologies shared between reconstruction models. Although there was no arbitrary classification, no TRW chronologies were selected for use in both TEHD and CGT reconstructions, possibly due to the low correlation between the TEHD and CGT indices (*r* = −0.165 for 1958–2022). Tables S1 and S3 summarize TRW chronologies used for the reconstructions.

In the construction of reconstruction models for the TEHD index, a nested principal components regression (PCR) method was used, which has been widely used in various dendroclimatological studies (*7*, *12*, *19*, *43*, *44*, *64*). This approach efficiently extracts common signals retained in various TRW chronologies and maximizes the utilization of available data extending backward in time. For each nested step, TRW chronologies were *z* score standardized over a common period, followed by a principal components analysis. PCs with eigenvalues exceeding 1.0, in line with the Kaiser-Guttman rule (*90*), and having absolute correlations with the TEHD index above 0.20, were selected as predictors for the multiple linear regression (MLR) model. This selection criterion enabled the use of PCs as meaningful signals from the TEHD pattern, even if they did not account for a meaningful portion of the total data variance. To mitigate overfitting problems in the regression models, independent variables for the MLR models were limited to a maximum of four (refer to tables S4 and S5). The model used in this study followed the “one in ten rule” (*91*), which suggests that one predictor variable can be included in a model for every 10 events (i.e., four variables for 43 calibration years). After constructing the initial nested reconstruction, the shortest TRW chronology was removed, and a new PCR model was developed. This process was repeated to build successive layers or “nests” of the model. To compile a comprehensive, full-length reconstruction, the time series outputs from each nest were merged. Before merging, we standardized the mean and variance of each nested time series to match those of the most replicated nest. This standardization was crucial to mitigate potential biases arising from the decreasing number of available TRW chronologies in earlier periods. Using this methodology, we reconstructed the TEHD index on an above-interannual timescale from 1741 to 2000 across six nests and on an interannual timescale from 1674 to 2000 across six nests (table S4). The final TEHD index reconstruction for the common period (1741–2000) was produced by aggregating these individual reconstructions. Similarly, for the CGT index, the final reconstruction spanned from 1708 to 2000. On the interannual timescale, this covered six nests for the same period, while on the above-interannual timescale, it included three nests covering the period from 1674 to 2000 (table S5).

To ensure the robustness of our reconstruction models, specifically the MLR models, we conducted a split calibration and verification test. The TEHD index and TRW chronologies shared a timeframe from 1958 to 2000, which we divided into two periods: 1958–1978 and 1980–2000. We used the former period as the calibration period and the latter as the verification period. We then swapped the calibration and verification periods. We assessed the accuracy of reconstruction with several statistical measures. During the calibration period, we evaluated the model’s skill using the Pearson correlation (*r*), adjusted coefficient of multiple determination ($R_{\text{adj}}^{2}$), and reduction of error (RE) statistic. In the verification period, we used the squared Pearson correlation coefficient (*R*^2^) and the coefficient of efficiency (CE)—see Cook *et al.* (*92*) for a full explanation of these statistics. In addition, we measured uncertainty using the root mean square error (RMSE), presented as ±1 RMSE. The performance of these metrics across all nested subsets of the model confirmed the credibility of our reconstruction models, as detailed in figs. S4 and S7.

### Wave activity flux

To describe the horizontal propagation of Rossby energy caused by Atlantic oceanic warming and African deep monsoonal convection strengthened in recent decades, we calculated the horizontal component of WAF (***W***) using Eq. 1 (*93*). The given diagnostic tool is useful for illustrating a snapshot of a propagating packet of stationary or migratory quasi-geostrophic wave disturbances and thereby for inferring where the packet is emitted and absorbed [see (*93*) for a detailed description].

$$W=\frac{p\text{cos}\phi }{2|U|}\left\{ \begin{array}{c} \frac{U}{a^{2}{\text{cos}}^{2}\varphi }\left[{\left(\frac{{\partial \psi }^{\prime }}{\partial \lambda }\right)}^{2}-\psi \prime \frac{\partial^{2}\psi \prime }{\partial \lambda^{2}}\right]+\frac{V}{a^{2}\text{cos}\varphi }\left[\frac{\partial \psi \prime }{\partial \lambda }\frac{\partial \psi \prime }{\partial \varphi }-\psi \prime \frac{\partial^{2}\psi \prime }{\partial \lambda \partial \varphi }\right]\\ \frac{U}{a^{2}\text{cos}\varphi }\left[\frac{\partial \psi \prime }{\partial \lambda }\frac{\partial \psi \prime }{\partial \varphi }-\psi \prime \frac{\partial^{2}\psi \prime }{\partial \lambda \partial \varphi }\right]+\frac{V}{a^{2}}\left[{\left(\frac{{\partial \psi }^{\prime }}{\partial \varphi }\right)}^{2}-\psi \prime \frac{\partial^{2}\psi \prime }{\partial \varphi^{2}}\right] \end{array}\right.$$ (1)

In Eq. 1, *p* is the unitless pressure (pressure/1000 hPa), the basic flow is denoted by $U={\left(U,V\right)}^{T}$, in which *U* and *V* are the climatological mean zonal and meridional winds, respectively, *a* is Earth’s radius, φ and λ are the latitude and longitude, respectively, and ψ′ is the stream function anomalies. ψ′ can be calculated by multiplying geopotential height anomalies by gravitational acceleration and dividing by Coriolis parameter. The WAFs over the regions where climatologically easterly wind prevails were excluded (*93*).

### Land-atmosphere coupling strengths

Miralles *et al.* (*23*) proposed a metric, π (Eq. 2), to assess the strength of land-atmosphere coupling strength. This metric is calculated as the product of the daily anomalies in near-surface air temperature (*T*′) and the change in surface energy balance depending on the degree of soil moisture deficits (*e*′) (*17*). The latter term becomes zero when soil moisture is sufficient to meet the atmospheric demand of evaporation, increasing under dry conditions as the atmospheric demand escalates. It can be calculated as Eq. 3, where *R*_n_ refers to the surface net radiation, λ*E* is the latent heat flux, and λ*E*_p_ is the latent heat flux based on potential evaporation (*E*_p_) instead of actual evaporation (*E*). The normalized anomalies, expressed as the number of SDs relative to the multiyear (1979–2022) mean for each day of the year, are indicated by primes. Daily climatological mean and variance were measured using a centered 15-day window. *T*, *R*_n_, and *E* were taken from the JRA-55 dataset. The latent heat of vaporization (λ) can be calculated as a function of *T*, and *E*_p_ can be estimated as a function of *T* and *R*_n_ (*94*). Values of π less than or equal to zero indicate no coupling, while higher values indicate stronger coupling.

π=T′*e′ (2)

$$e\prime =(R_{n}-\lambda E)\prime -(R_{n}-\lambda E_{p})\prime$$ (3)

### CMIP6 climate simulations

The study analyzed the simulation results from 21 climate models that participated in the CMIP6 (table S6) (*71*). The models were selected on the basis of the availability of the required variables: monthly mean geopotential height in a historical scenario and the SSP future scenarios (SSP126, SSP245, SSP370, and SSP585), daily maximum SAT and monthly mean SAT, and total precipitation in the historical scenario. Although some models have several ensemble members, only the first ensemble, which variant label is “r1i1p1f1,” for each simulation was analyzed to ensure equal weighting. Before analysis, all model outputs were bilinearly interpolated into longitudinal and latitudinal 1.25° resolution, which is consistent with JRA-55.

As the CMIP6 historical simulation covers the period from 1850 to 2014, climatological base period was set to 1979–2014 not 1979–2022. Heatwave characteristics were also calculated from the base period 1979–2014. For drought characteristics, SPEI values were calculated from monthly mean SAT and precipitation. In the observation dataset, potential evapotranspiration was estimated using the FAO-56 (Food and Agriculture Organization of the United Nations Irrigation and Drainage Paper No. 56) Penman-Monteith method (*95*), and SPEI was calculated by fitting a log-logistic distribution to the difference between the precipitation and potential evapotranspiration. However, in the CMIP6 dataset, potential evapotranspiration was estimated using the Thornthwaite method (*96*), and the difference between potential evapotranspiration and precipitation was modeled using a Pearson III distribution. This approach was chosen to simplify calculations and make use of as much model output as possible.

## Supplementary Materials

### This PDF file includes:

Figs. S1 to S17 Tables S1 to S6 References
