# Supplementary material for: Emerging trans-Eurasian heatwave-drought train in a warming climate
Source: Sci Adv. 2025 May 2;11(18):eadr7320. doi: 10.1126/sciadv.adr7320 (PMC12047423; doi:10.1126/sciadv.adr7320)
Supplement: Supplementary file 1 — Figs. S1 to S17 Tables S1 to S6 References [file sciadv.adr7320_sm.pdf]

Science Advances
AAAS

Supplementary Materials for

Emerging trans-Eurasian heatwave-drought train in a warming climate

Jee-Hoon Jeong *et al.*

Corresponding author: Jee-Hoon Jeong, jjeehoon@sejong.ac.kr; Min-Seok Kim, minseok7kim@gmail.com

Sci. Adv. **11**, eadr7320 (2025)
DOI: 10.1126/sciadv.adr7320

This PDF file includes:

Figs. S1 to S17
Tables S1 to S6
References

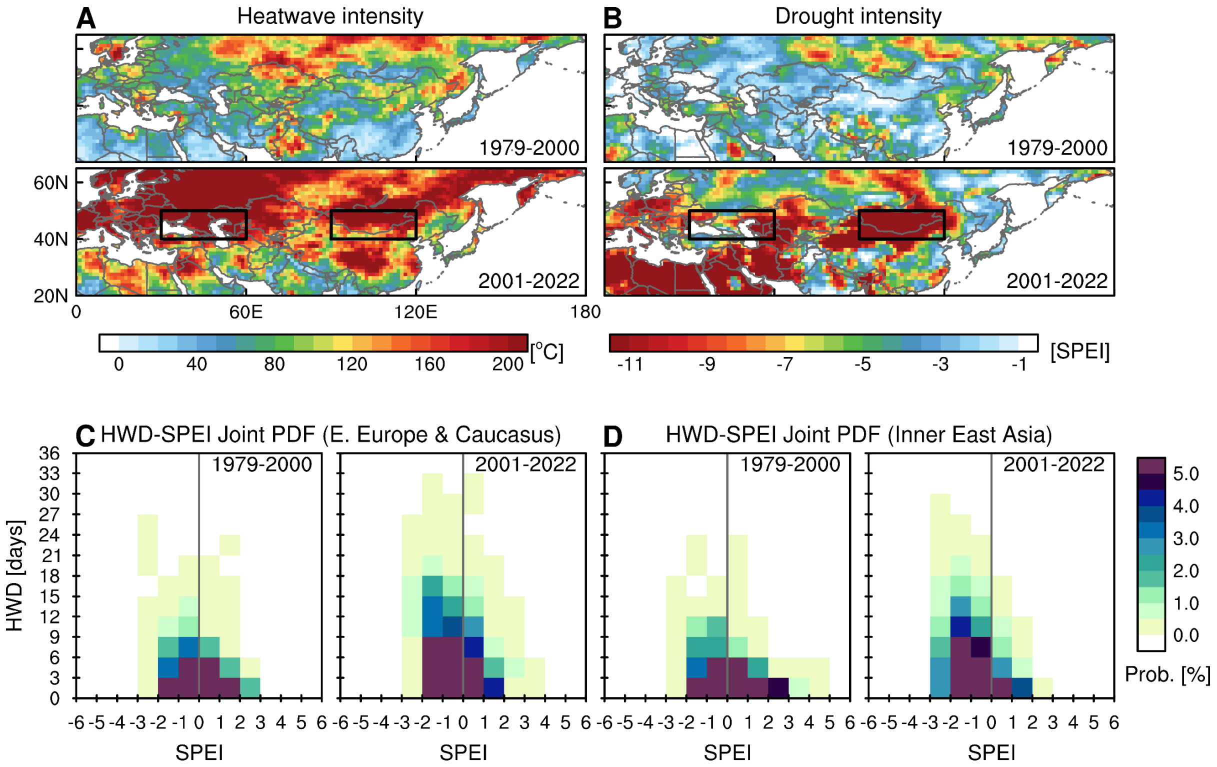

Fig. S1. Recent changes in heatwave-drought intensity and coupling over Eurasia. (**A** and **B**) Cumulative heatwave and drought intensity in 1979-2000 and 2001-2022. Cumulative heatwave intensity is the sum of the temperature anomaly between each heatwave day and the extreme heatwave threshold (i.e. 90th percentile) across all heatwave days in that summer following Perkins-Kirkpatrick and Lewis (*75*). Cumulative drought intensity was calculated in the same way using SPEI anomalies. (**C** and **D**) Heatwave-drought coupling strength changes represented by the joint probability density function of JA total heatwave days and mean SPEI6 over Eastern Europe and Caucasus (C) and Inner East Asia (D) in 1979-2000 and 2001-2022. The solid black frames in (A) and (B) represent the domain, Eastern Europe and Caucasus (left, 40-50°N, 30-60°E) and Inner East Asia (right, 40-50°N, 90-120°E), respectively.

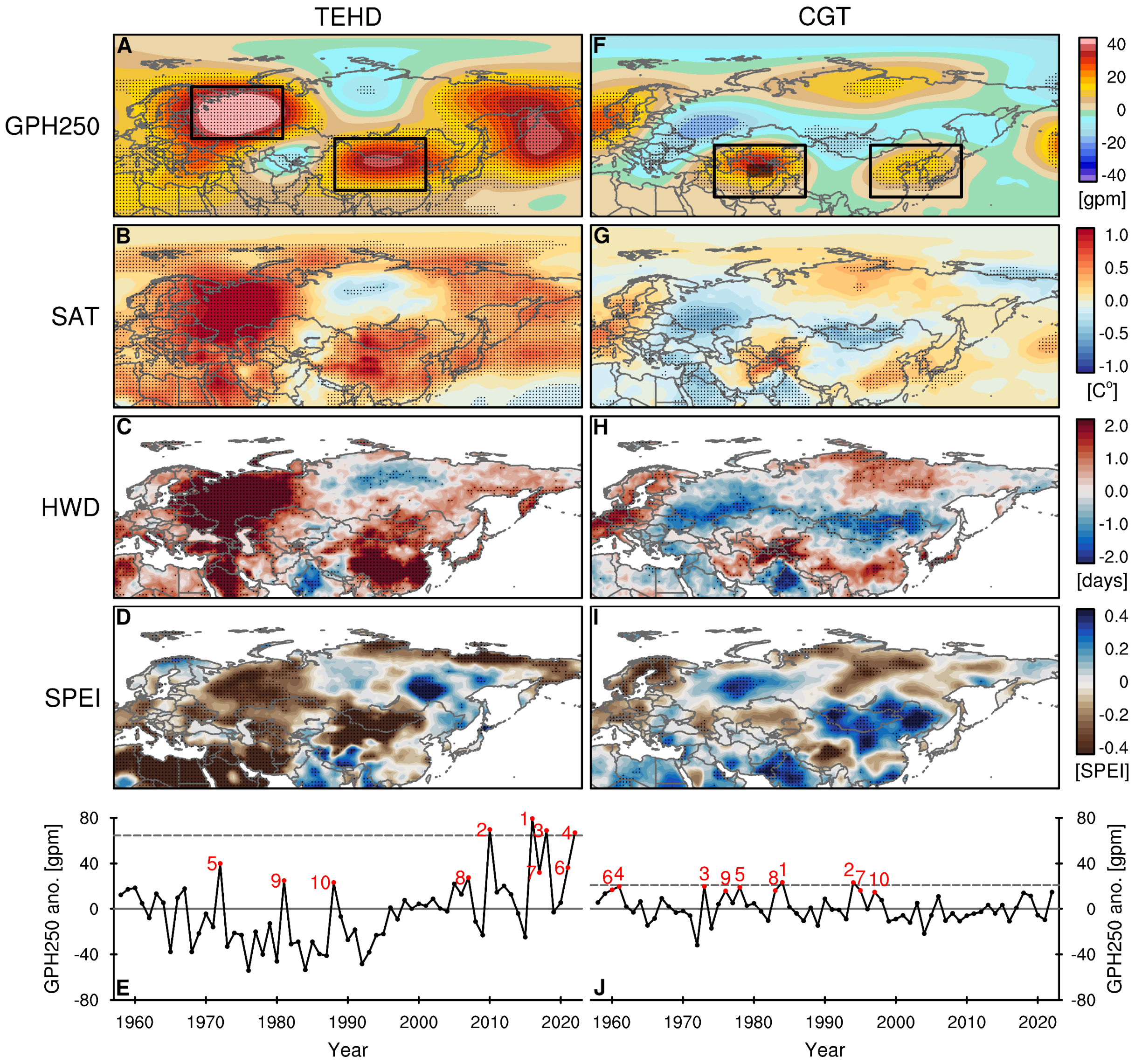

Fig. S2. Spatial patterns and recent temporal variations of TEHD and CGT. (**A** to **D**) Regression coefficients of JA mean GPH250 (A), mean SAT (B), total HWD (C), and mean SPEI6 (D) anomalies with respect to Z-score standardized TEHD index in 1979-2022. Black dots denote statistically significant regression coefficients at the $p < 0.10$ level. (**E**) The TEHD index in 1958-2022. (**F** to **J**) same as (A to E) but for the CGT index. In (E) and (J), red dots with numbers denote the top 10 strongest TEHD and CGT years. Grey solid and dashed lines represent the mean and 2σ of each index in 1979-2022, respectively.

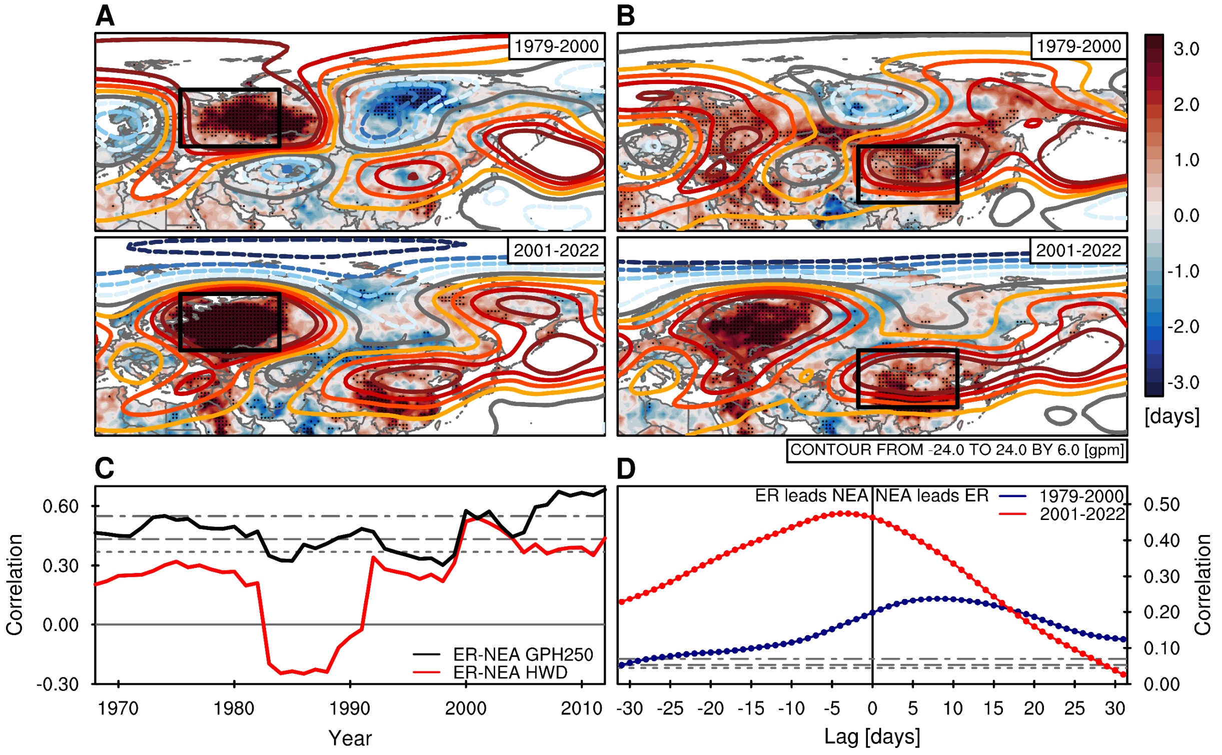

Fig. S3. Recent changes in correlation between the TEHD core regions and their relationship with GPH250 and HWD anomalies. (**A**) Regression coefficients of JA mean GPH250 (contour) and total HWD (shading) anomalies with respect to JA mean GPH250 anomalies (Z-score standardized for 1979-2022) averaged over European Russia (black frame; 50-70°N, 30-65°E) in 1979-2000 (upper) and 2001-2022 (lower). Black dots denote the grids where HWD has statistically significant ($p < 0.10$) regression with the averaged GPH250 anomalies. (**B**) Same as (A) but for the regression coefficients respect to the GPH250 anomalies averaged over Northeast Asia (30-50°N, 85-120°E). (**C**) Running correlation coefficients on 21-year window (e.g., the correlation in 2000 represents the period 1990-2010) between JA GPH250 (black) anomalies averaged over European Russia and Northeast Asia. Red line represents the running correlation of JA HWD anomalies averaged over the two domains. (**D**) Daily lead-lag correlation between moving 21-day-mean GPH250 anomalies averaged over European Russia and Northeast Asia during JA in 1979-2000 (blue) and 2001-2022 (red). In (c) and (d), grey dashed, dotted, and dash-dotted lines represent the level of statistical significance at the $p < 0.10$, 0.05, and 0.01, respectively.

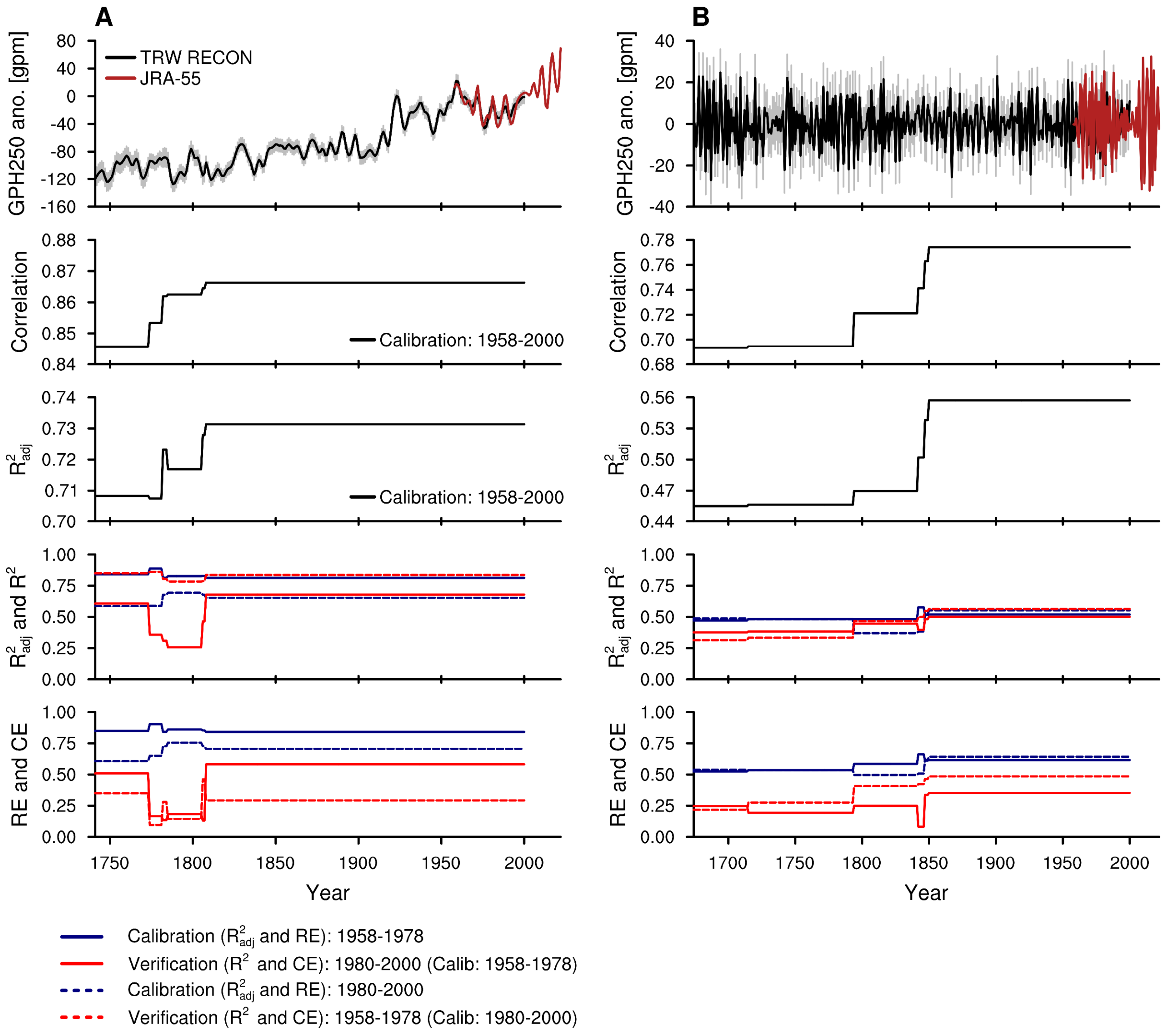

Fig. S4. Reconstruction of the TEHD index based on the TRW chronologies. (**A** and **B**) Reconstructions of the TEHD index on above-interannual (A) and interannual (B) timescales with reconstruction skill scores.

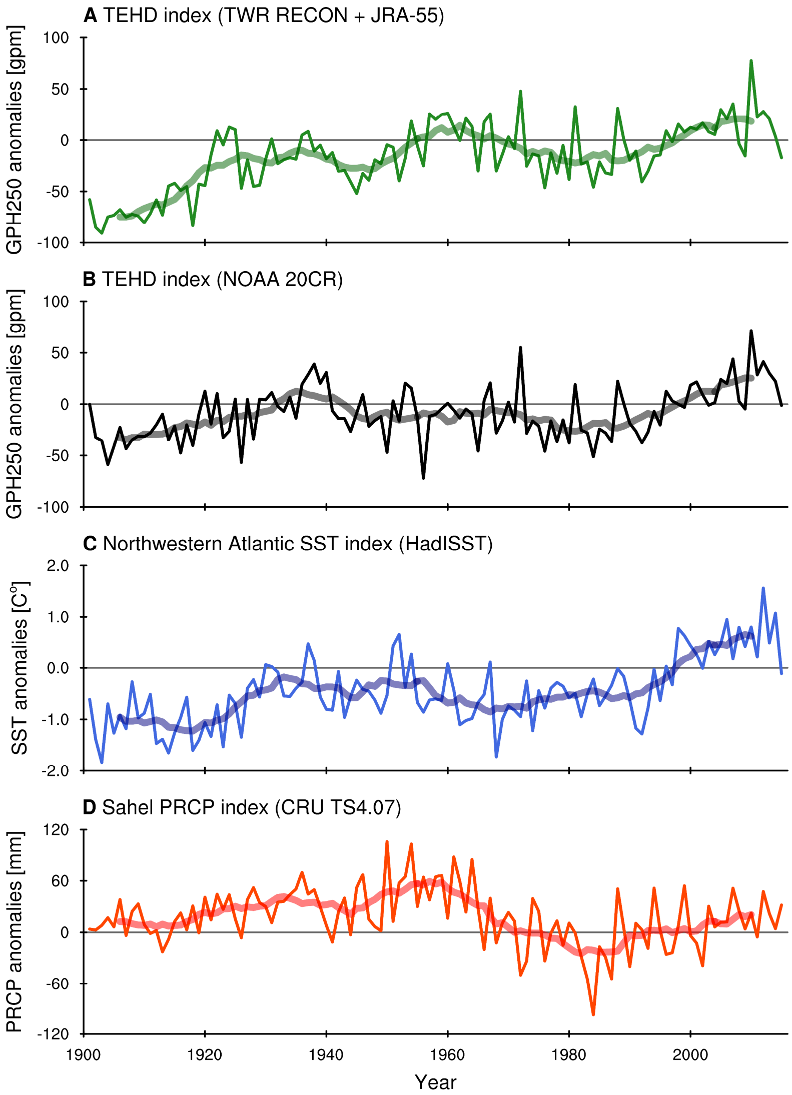

Fig. S5. Temporal variation of the TEHD and its forcing factors over the 20th century. (**A**) TEHD index derived from tree-ring reconstruction for the period 1901-1957 and from the JRA-55 for the period 1958-2015. (**B**) TEHD index obtained from the National Oceanic and Atmospheric Administration's 20th Century Reanalysis (NOAA 20CR) for the period 1901-2015. (**C**) JA Northwestern Atlantic SST anomalies from the HadISST dataset for the period 1901-2015. (**D**) JA Sahel precipitation anomalies from the CRU TS version 4.07 dataset for the period 1901-2015. Translucent lines indicate 11-year running means.

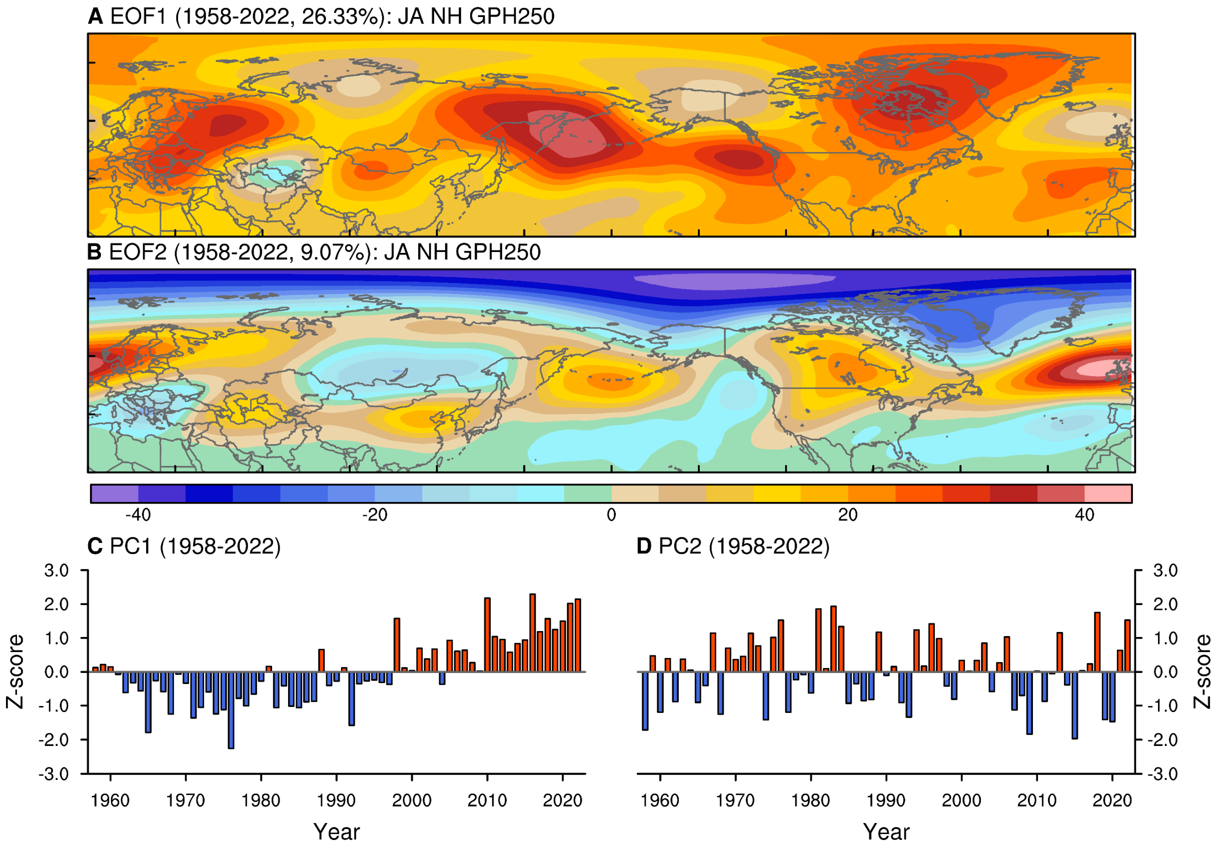

Fig. S6. Leading modes of the Northern Hemisphere upper-atmospheric circulation in summer. The first (**A**) and second (**B**) EOF modes of the JA mean GPH250 anomalies over the Northern Hemisphere for the period 1958-2022, along with their corresponding PC time series (**C** and **D**).

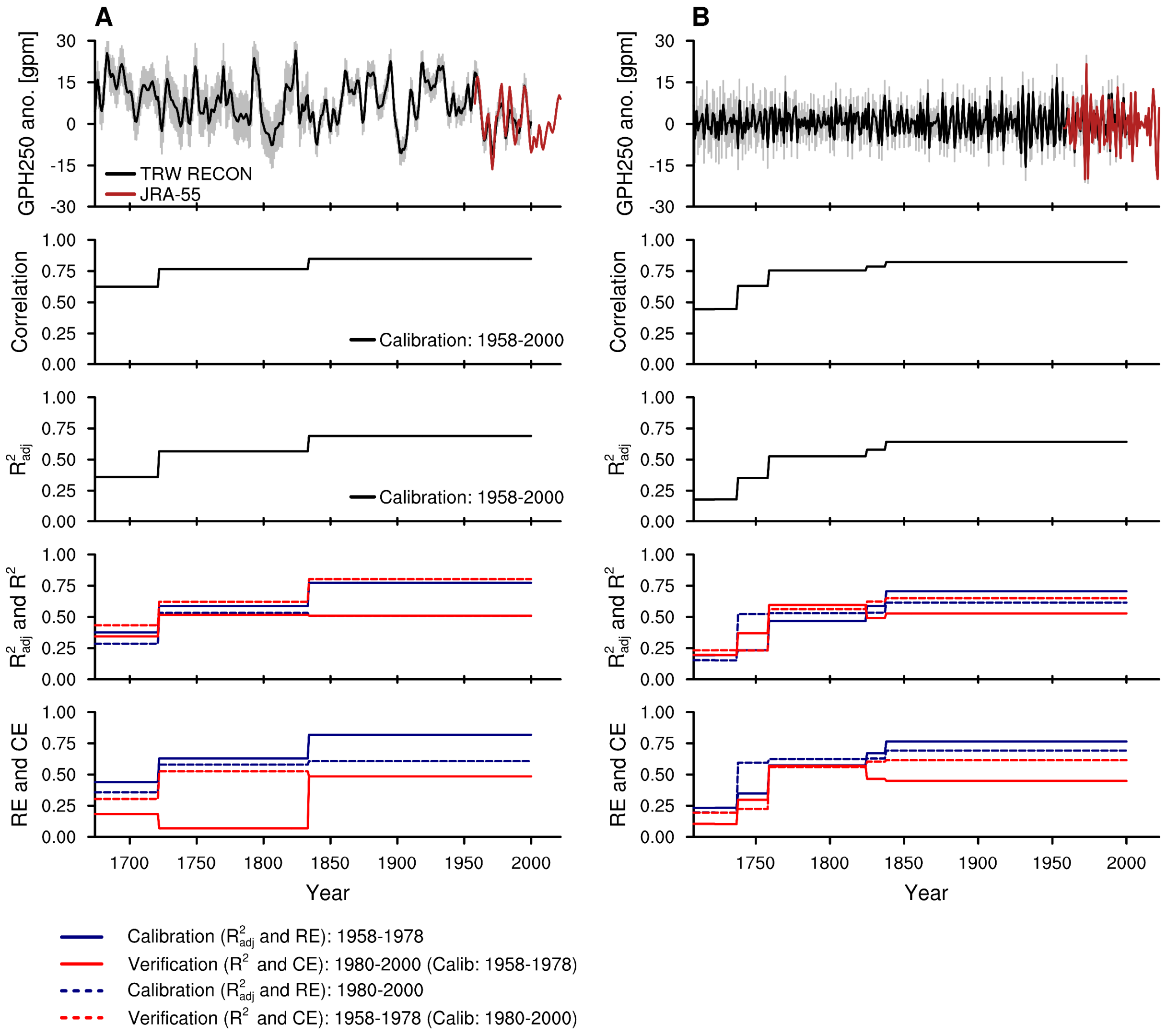

Fig. S7. Reconstruction of the CGT index based on the TRW chronologies. Same as fig. S4 but for the CGT index reconstructions.

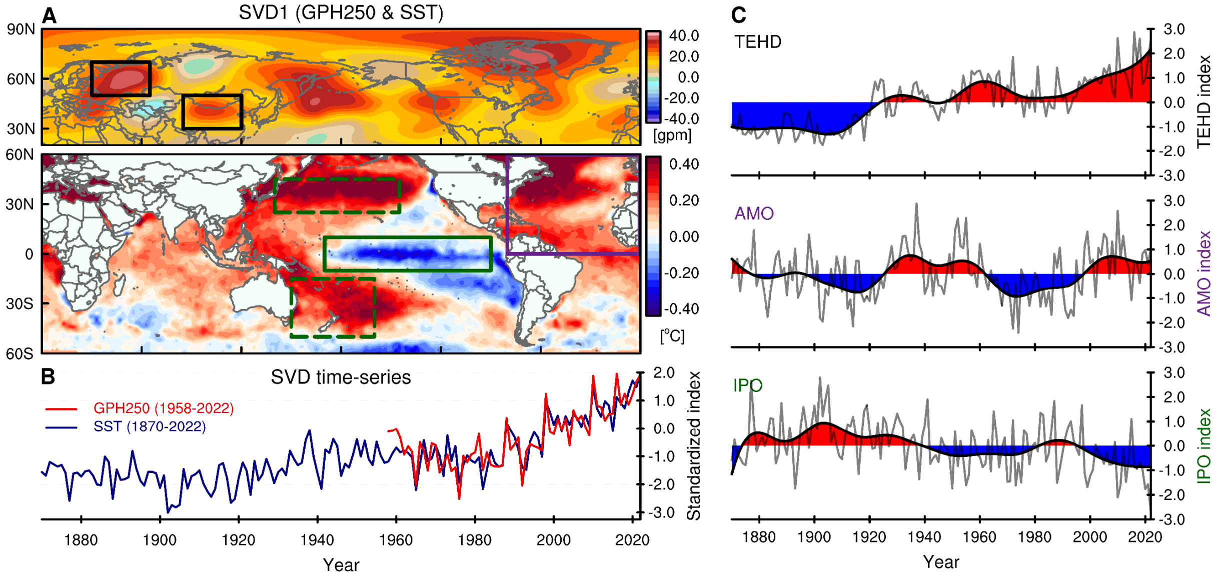

Fig. S8. Leading coupled pattern of the Northern Hemisphere upper-atmospheric circulation and global SST in summer. (**A**) The first leading singular value decomposition (SVD) mode of the JA mean GPH250 and SST anomalies for the period 1979-2022. The percentage of the squared covariance fraction of the mode is 64.40%. (**B**) The time-series of the first leading SVD mode constructed by projecting the JA mean GPH250 (red) and SST (blue) anomalies onto the loading pattern. Both time-series are Z-score standardized for 1979-2022. (**C**) The TEHD (1870-1957: reconstruction, 1958-2022: JRA-55; top) and AMO (Atlantic Multidecadal Oscillation; middle) and IPO (Interdecadal Pacific Oscillation; bottom) indexes for the period 1870-2022. The AMO index is defined as the JA SST anomalies averaged over the North Atlantic (0-60°N, 0-80°E; purple solid frame in A) (*97*). The IPO index is defined as the difference between the SST anomalies averaged over the central equatorial Pacific (10°S-10°N, 170-270°E, green solid frame in A) and the average of the SST anomalies in the Northwest (25-45°N, 140-215°E) and Southwest (15-50°S, 150-200°E) Pacific (green dashed frames in A) (*98*). The global warming signal was removed by subtracting the global mean (60°S-60°N, 0-360°E) SST anomalies from both the AMO and IPO indexes (*97*).

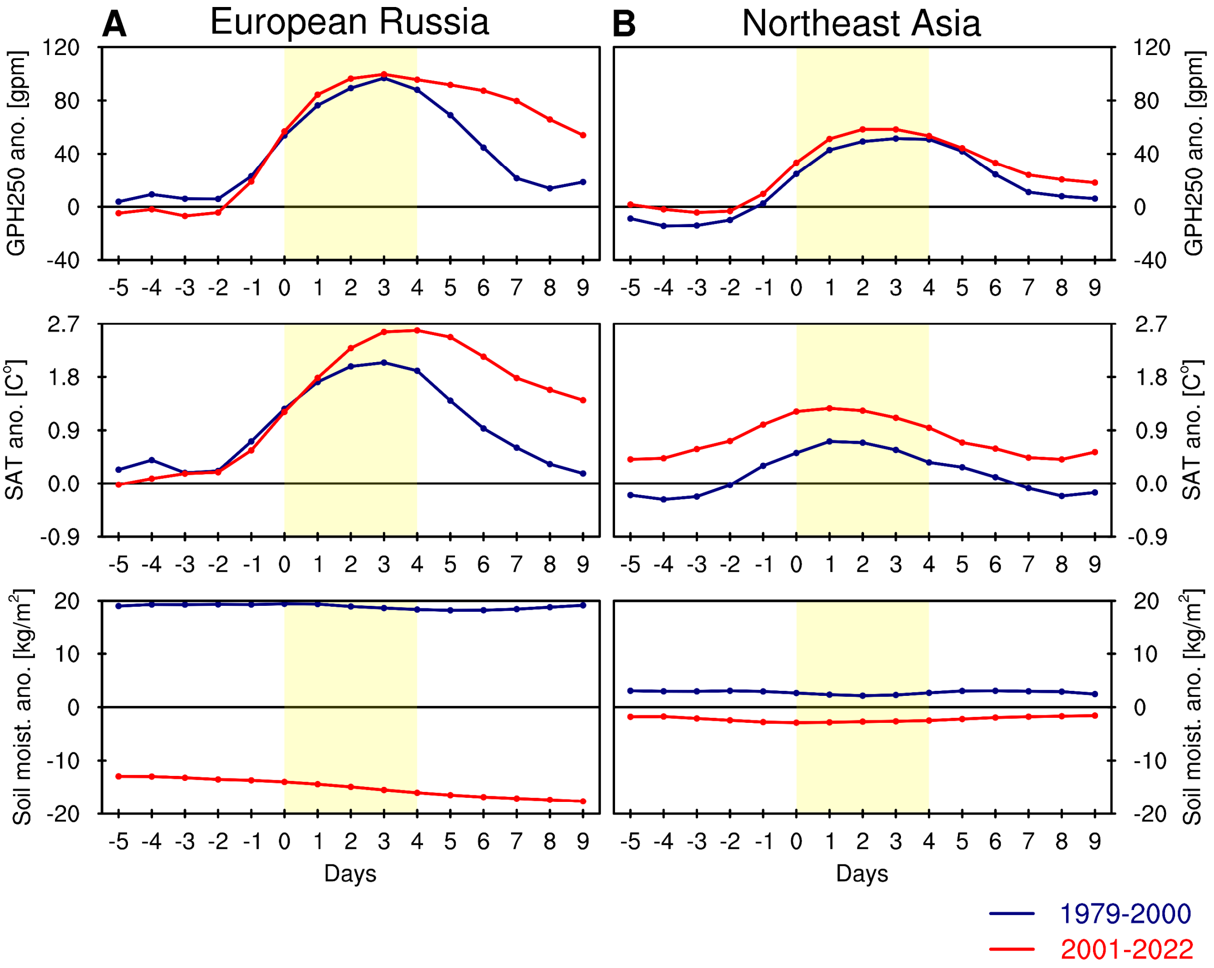

Fig. S9. Daily evolution of the blocking high events over the TEHD core regions before and after 2000. (**A** and **B**) Daily composite of GPH250 and SAT and soil moisture anomalies averaged over European Russia (A, left frame in Fig. 2A) and Northeast Asia (B, right frame in Fig. 2A) from 5 days before the onset of the blocking high event to 9 days after that in 1979-2000 and 2001-2022. Day 0 indicates the onset day of the blocking high event.

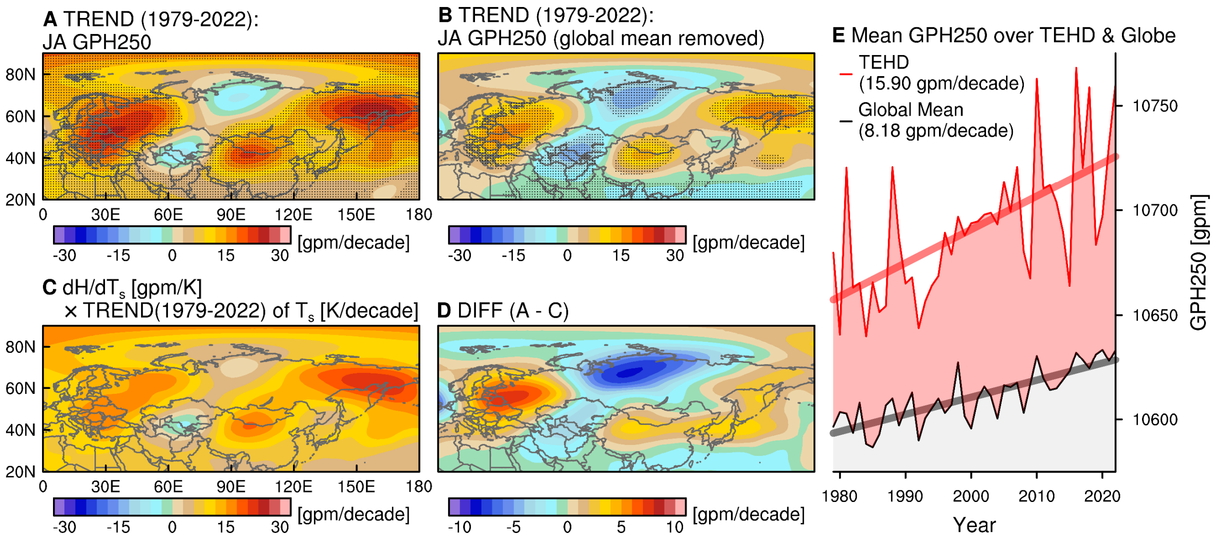

Fig. S10. Recent TEHD amplification trends exceeding the global mean GPH increasing trends. (**A**) Linear trends of JA GPH250 in 1979-2022. (**B**) Linear trends of global-mean-removed JA GPH250 in 1979-2022. In (A) and (B), crosses represent regions where trends are statistically significant at the $p < 0.10$ level. (**C**) Map of GPH250 sensitivity to global mean surface air temperature (dH/dT_s; (*99*)) (units: gpm/°C), multiplied by the trend of global mean surface air temperature (T_s, units: °C/decade). (**D**) Difference between panels A and C (A minus C). (**E**) Time series of JA GPH250 averaged over the TEHD core regions (European Russia and Northeast Asia) (red line) and global mean (black line) in 1979-2022. Thick lines indicate linear trends, and numbers in parentheses denote trend values.

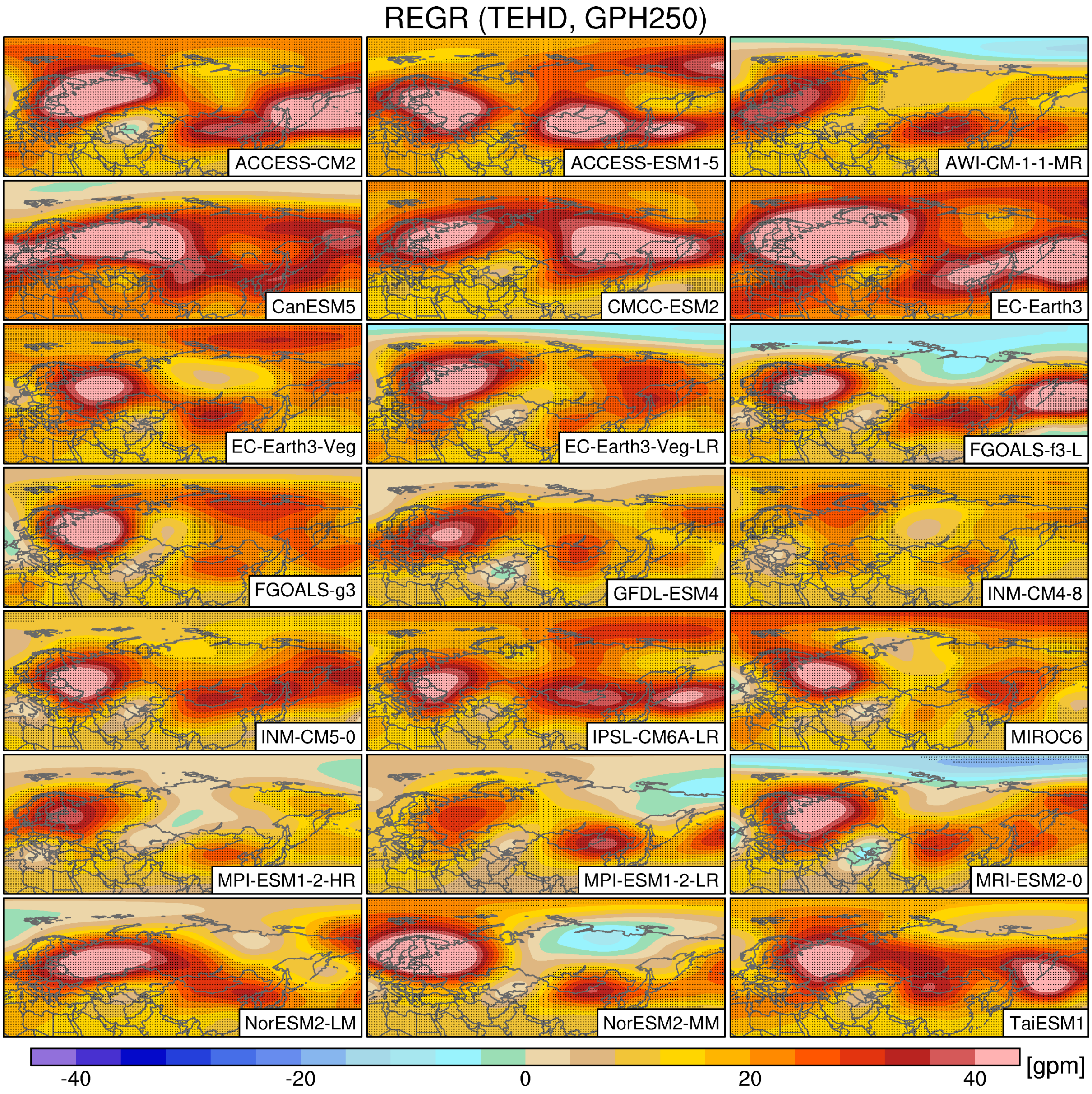

Fig. S11. Simulated TEHD patterns in 21 CMIP6 models. Regression coefficients of JA GPH250 anomalies with respect to Z-score standardized TEHD index for the period 1979-2014 from the 21 CMIP6 models. Black dots denote statistically significant values at the $p < 0.10$ level.

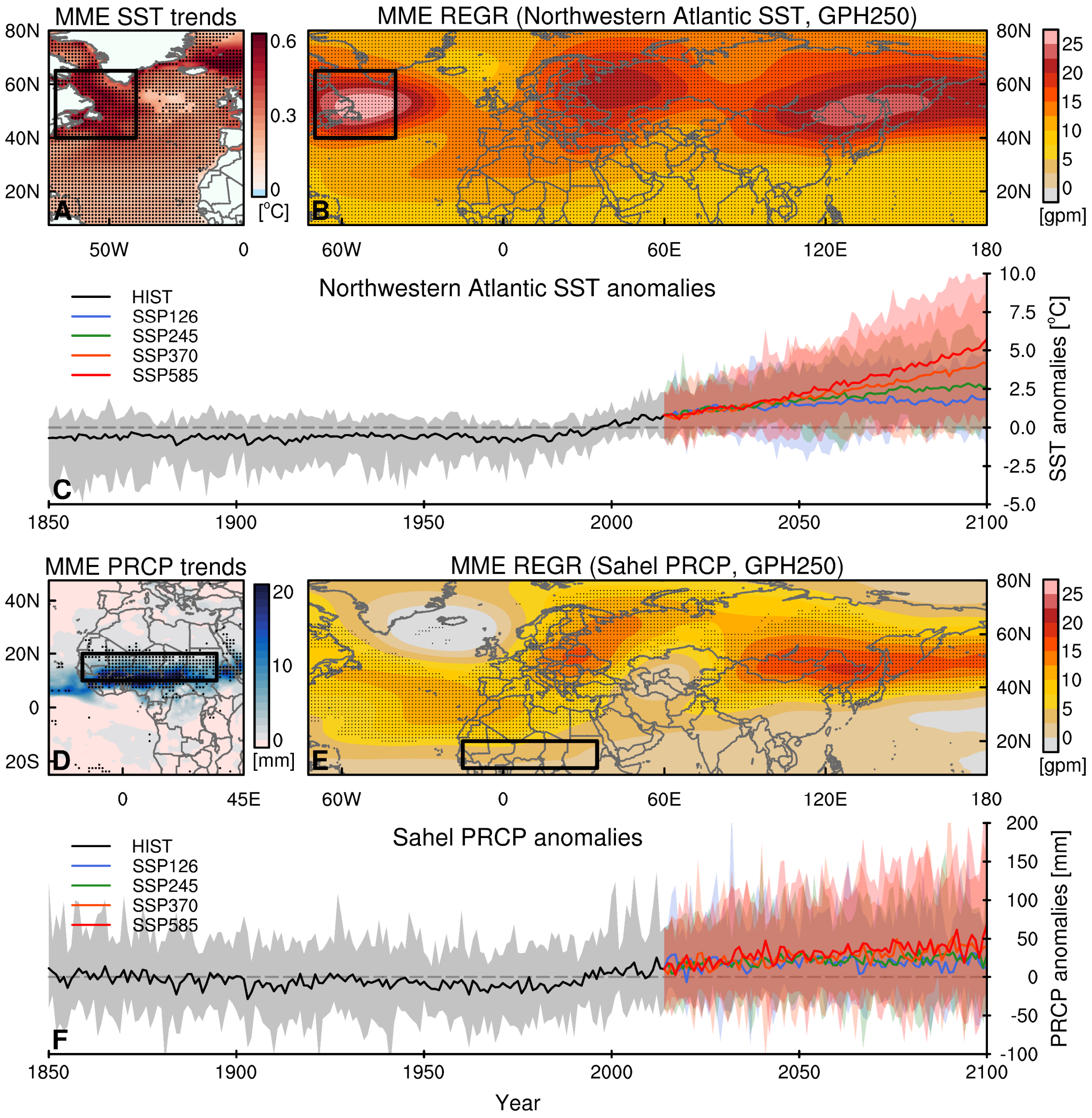

Fig. S12. TEHD strengthening mechanisms in the CMIP6 models. (**A** and **B**) MME linear trends of JA SST (A) and regression coefficients of JA GPH250 anomalies with respect to Z-score standardized JA SST anomalies averaged over the Northwestern Atlantic (40-65°N, 40-70°W) in 1979-2014 (B) from the 21 CMIP6 models. (**C**) MME JA mean SST anomalies averaged over the Northwestern Atlantic in the historical simulation (black) and the future projections based on the four SSP scenarios from the 21 CMIP6 models. Shaded area represents the ensemble spread of the models. (**D** to **F**) Same as (A to C), but for the total precipitation changes over the African Sahel (10-20°N, 15°W-35°E). In (A), (B), (D), and (E), black dots denote the grids where 17 or more models show the same sign as the MME response.

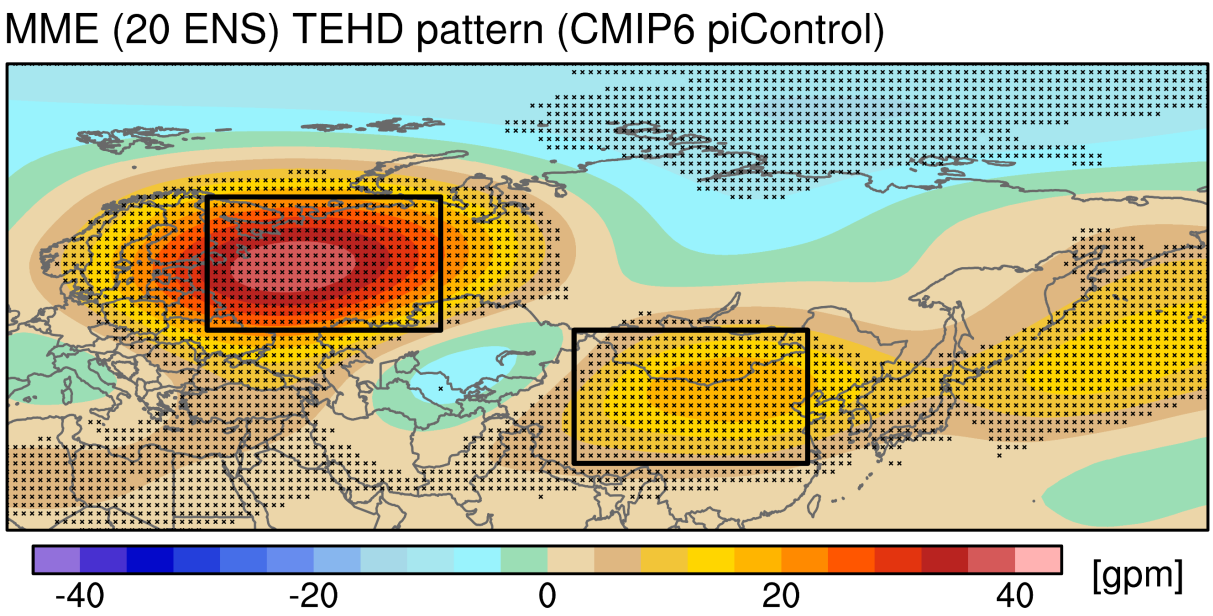

Fig. S13. TEHD pattern in the CMIP6 piControl experiment. MME (20 models) TEHD patten in CMIP6 piControl experiments. The TEHD patten represented as regression coefficients of JA GPH250 anomalies with respect to the Z-score standardized TEHD index for the first 30 year simulation in each model simulation. Twenty models used are denoted in fig. S14. Crosses represent regions where 16 or more models (about 80% of the total number of models) show the same sign with the MME response.

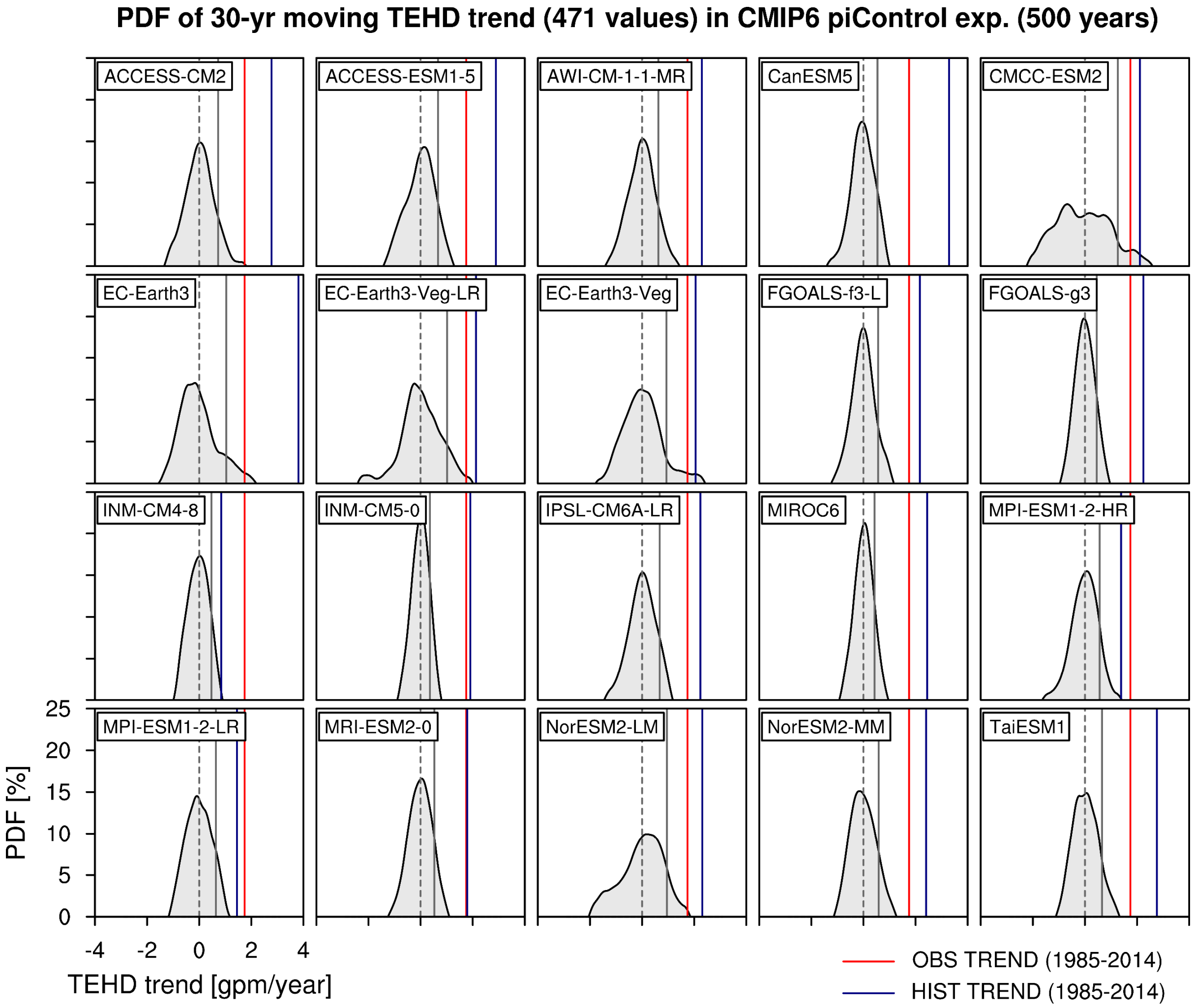

Fig. S14. Comparison of recent TEHD trends with CMIP6 piControl simulations. The probability distribution function (PDF) of moving 30-year trends of the TEHD index in CMIP6 pre-industrial control (piControl) 500-year simulations (distribution of 471 trend values). The vertical dotted grey line represents the zero value, and the vertical solid grey lines represent the mean +2 standard deviations. The vertical solid blue line represents the trend of the TEHD index for the period 1985-2014 in CMIP6 historical simulations, while the vertical solid orange line represents the trend of the TEHD index for the same period in the JRA-55.

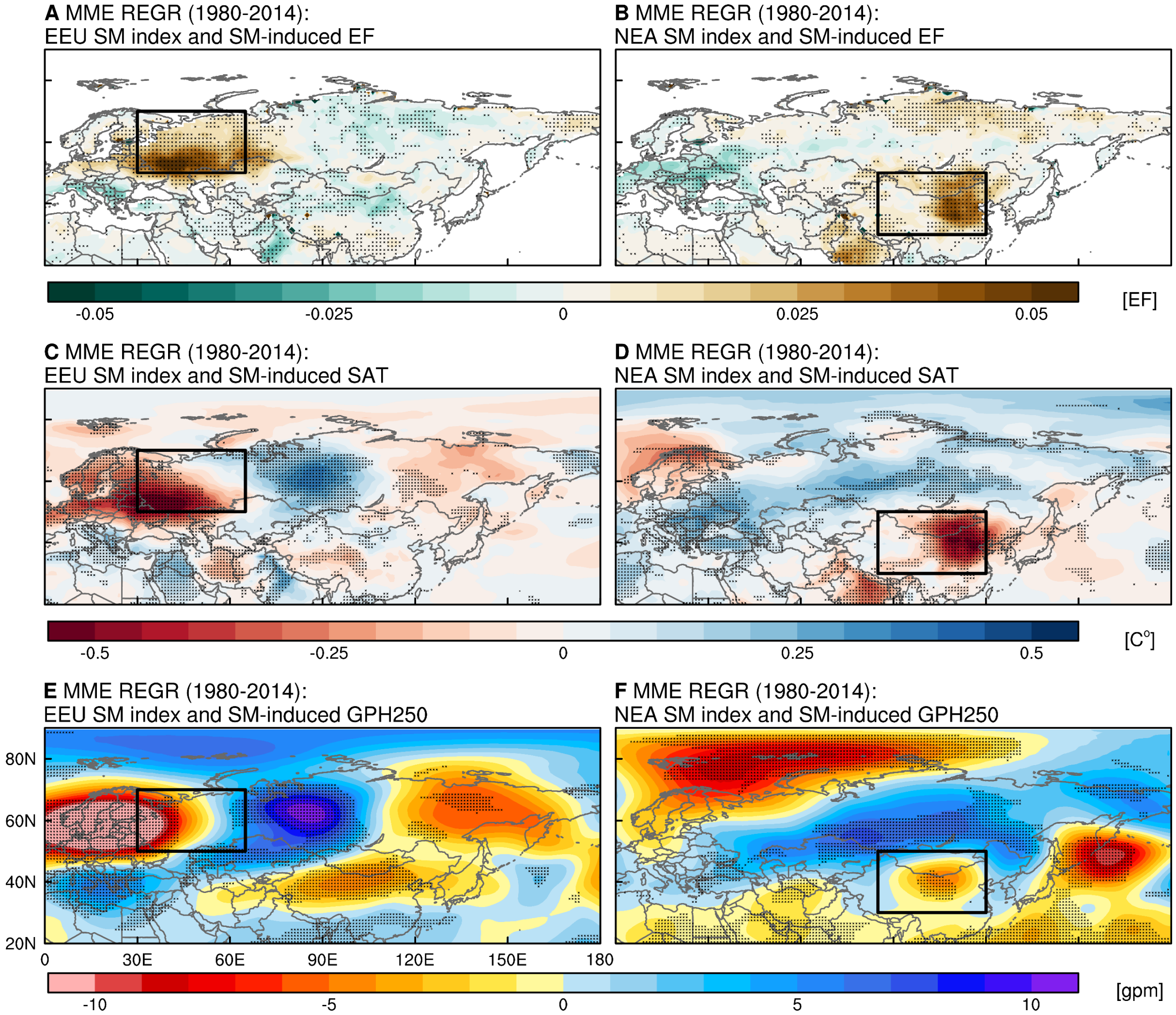

Fig. S15. Land-atmosphere responses to soil moisture variation over the TEHD core regions. (**A, C,** and **E**) MME (6 models) regression coefficients of soil moisture-induced variations in July-August (JA) evaporative fraction (EF; A), surface air temperature (SAT; C), and 250 hPa geopotential height (GPH250; E) with respect to soil moisture anomalies (from CMIP6 historical simulations) averaged over European Russia for the period 1980-2014. Soil moisture-induced changes in EF, SAT, and GPH250 represent the difference between CMIP6 historical simulations and CMIP6 LFMIP-pdLC simulations. The LFMIP-pdLC experiment uses fixed modern-day land surface conditions (climatological monthly mean from CMIP6 historical simulations in 1980-2014) throughout the simulation period; thus, the difference with CMIP6 historical simulations can represent the soil moisture effects on climate. (**B**, **D**, and **F**) Same as (A, C, and E) but for Northeast Asia. Crosses denote the grid points where about 80% of models ($\geq$ 5 models) exhibit the same MME sign. Six models used: CESM2, CMCC-ESM2, CNRM-CM6-1, EC-Earth3, IPSL-CM6A-LR, and MPI-ESM1-2-LR.

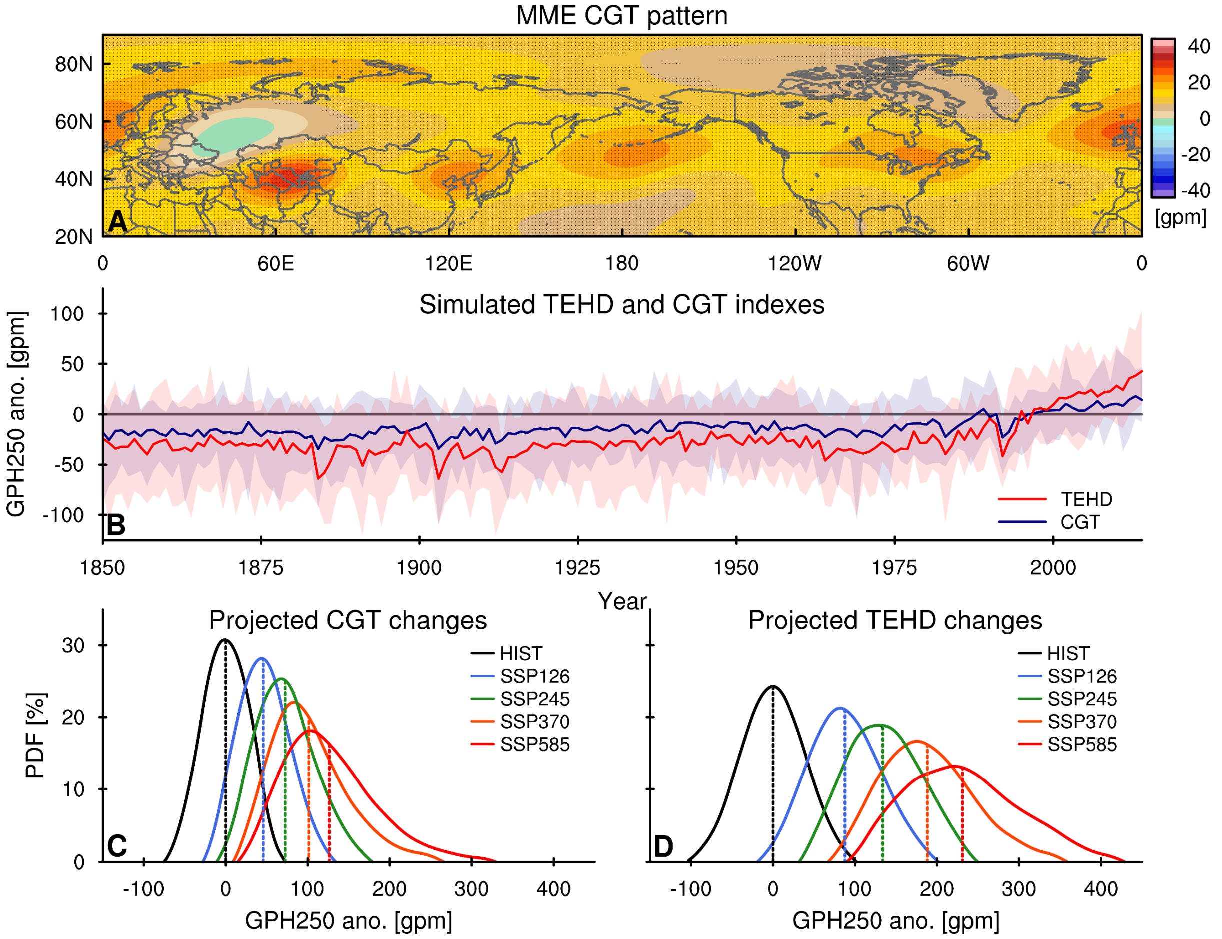

Fig. S16. Projected future changes of TEHD and CGT in CMIP6 models. (**A**) MME regression coefficients of JA GPH250 anomalies with respect to the Z-score standardized CGT index in 1979-2014 from the 21 CMIP6 models. Black dots denote the grids where 17 or more models show the same sign with the MME response. (**B**) MME TEHD and CGT indexes in the CMIP6 historical simulation. The shaded area indicates the ensemble spread of the 21 CMIP6 models. (**C** and **D**) The probability distribution function of the CGT index (C) and the TEHD index (D) in 1979-2014 (historical simulation) and 2065-2100 (future projections based on the four SSP scenarios.

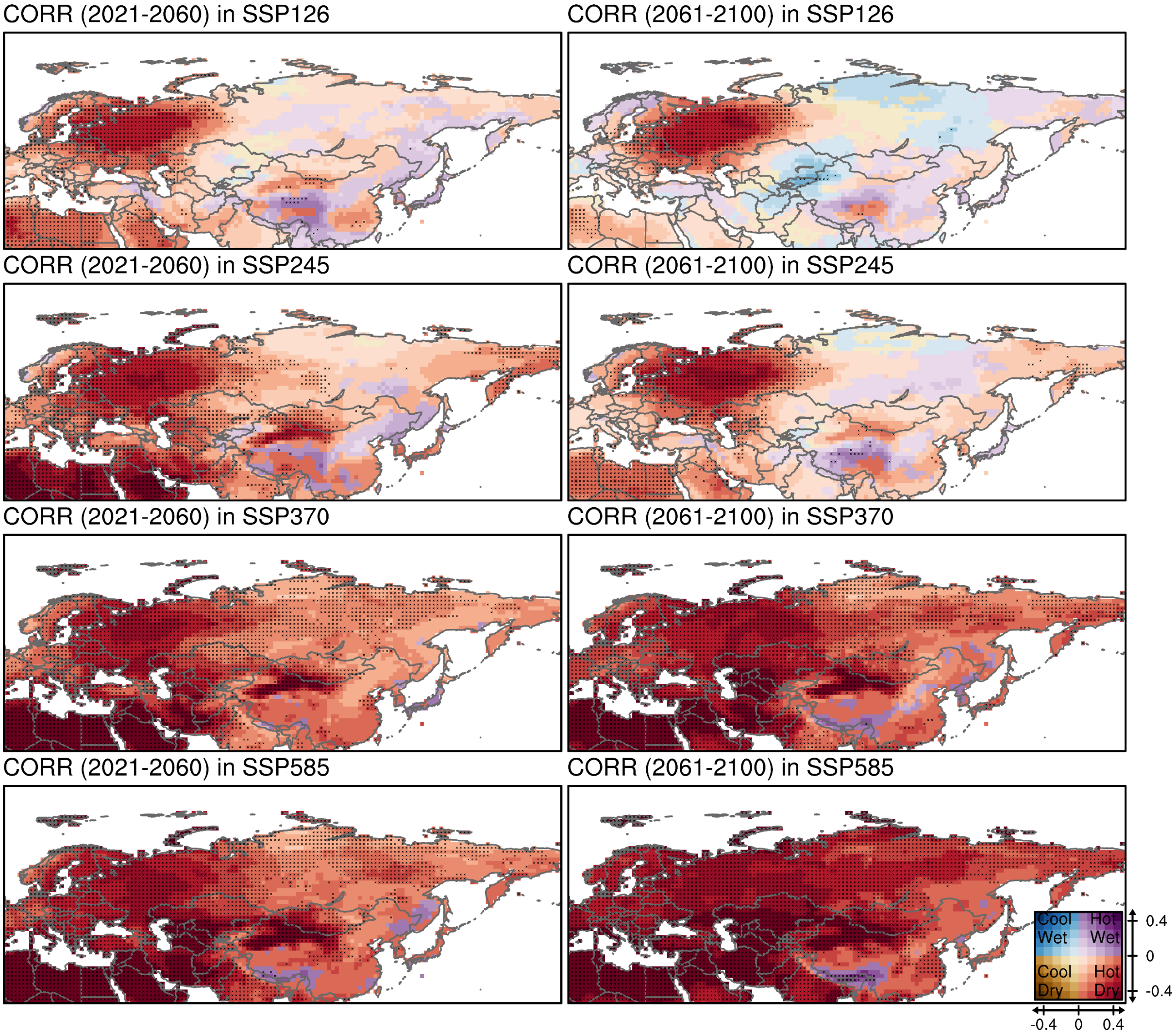

Fig. S17. Future impacts of the TEHD on heatwave-drought occurrences. CMIP6 MME (20 models) correlation patterns of JA HWD/SPEI6 with respect to z-score TEHD index in future. The mean values of 20 models, excluding model 'FGOALS-f3-L' out of the 21 models used in Fig. 5, are presented. Crosses represent regions where 16 or more models (about 80% of the total number of models) show the same sign with the MME response.

Table S1. Information of tree-ring data used in the TEHD index reconstruction.

No.	Name	Country	Lat. (°N)	Lon. (°E)	Elev. (m)	Species	Time span (CE)
Tree-ring data used in the TEHD index reconstruction for both above-interannual and interannual timescale							
1	CZEC016	Czech Republic	50.20	16.85	1300	PCAB	1741 - 2011
2	FINL076	Finland	68.93	28.42	150	PISY	1508 - 2003
3	POLA030	Poland	49.52	19.03	1300	PCAB	1627 - 2004
4	RUS318	Russia	65.18	35.97	10	PISY	1549 - 2011
5	RUSS183	Russia	64.77	32.33	N/A	PISY	1590 - 2002
6	RUSS264	Russia	55.96	160.23	944	LAGM	1653 - 2014
7	TURK016	Turkey	36.60	30.02	1853	JUEX	1332 - 2000
8	TURK034	Turkey	41.50	33.00	1050	QUSP	1607 - 2001
Tree-ring data used in the TEHD index reconstruction for above-interannual timescale							
9	AUST115	Austria	47.95	16.06	500	PILR	1319 - 2002
10	CHIN011	China	43.15	82.87	1499	PCSH	1634 - 2002
11	CHIN014	China	43.88	88.12	1913	PCSH	1694 - 2004
12	CHIN033	China	43.77	87.92	1970	PCSH	1653 - 2002
13	CHIN064	China	36.68	98.42	3700	JUPR	900 - 2001
14	FINL088	Finland	68.83	27.31	258	PISY	1511 - 2011
15	FINL096	Finland	68.77	27.15	170	PISY	1752 - 2015
16	GEOR002	Georgia	41.72	43.48	1850	PISY	1754 - 2006
17	KYRG015	Kyrgyzstan	42.15	79.45	3050	PCSH	1528 - 2005
18	MONG020	Mongolia	48.27	88.87	N/A	LASI	1537 - 2005
19	NORW007	Norway	69.42	25.63	350	PISY	1698 - 2001
20	POLA027	Poland	49.25	20.03	1480	PCAB	1628 - 2004
21	RUSS219	Russia	43.88	145.60	N/A	QUMO	1585 - 2000
22	SVK015	Slovakia	49.01	19.16	1100	PISY	1745 - 2018
23	SVK016	Slovakia	48.94	20.31	910	PISY	1696 - 2019
Tree-ring data used in the TEHD index reconstruction for interannual timescale							
24	CHIN018	China	29.28	100.08	4150	ABFO	1540 - 2006
25	PAKI031	Pakistan	35.50	74.08	N/A	CDDE	1296 - 2007
26	PAKI033	Pakistan	35.50	74.75	N/A	PIGE	1362 - 2007
27	POLA022	Poland	49.58	19.05	1300	PCAB	1687 - 2004
28	POLA025	Poland	49.25	20.03	1420	PCAB	1737 - 2004
29	RUS357	Russia	65.07	35.83	N/A	MIXD	1185 - 2008
30	RUSS209	Russia	55.00	160.50	N/A	LAGM	1776 - 2001
31	RUSS309	Russia	51.00	107.10	600	PISY	1760 - 2014
32	SVK013	Slovakia	48.99	19.22	1450	PCAB	1722 - 2018
33	TURK050	Turkey	39.88	29.83	1240	PINI	1721 - 2008

Note for table S1 and S3: ABFO = *Abies forestii Rogers*, CDDE = *Cedrus deodara (D. Don) G. Don*, FASY = *Fagus sylvatica L.*, JUEX = *Juniperus excelsa M.-Bieb.*, JUPR = *Juniperus przewalskii Komarov*, JUTI = *Juniperus tibetica Kom.*, LAGM = *Larix gmelinii (Rupr.) Kuzen.*, LALY = *Larix lyallii Parl.*, LASI = *Larix sibirica Ledeb.*, MIXD = Various taxa, PCAB = *Picea abies (L.) H. Karst.*, PCSH = *Picea schrenkiana Fisch. & C.A. Mey.*, PCSM = *Picea smithiana (Wall.) Boiss.*, PIED = *Pinus edulis Engelm.*, PIGE = *Pinus gerardiana Wall. ex D. Don.*, PILR = *Pinus laricio Poir.*, PINI = *Pinus nigra J.F. Arnold*, PIPO = *Pinus ponderosa Douglas ex C. Lawson*, PIST = *Pinus strobus L.*, PISY = *Pinus sylvestris L.*, PITB = *Pinus tabulaeformis Carr.*, PSME = *Pseudotsuga menziesii (Mirb.) Franco*, QUDG = *Quercus douglasii Hook. and Arn.*, QUMO = *Quercus mongolica Fisch. ex Turcz.*, QUPR = *Quercus prinus L.*, QURO = *Quercus robur L.*, QUSP = *Quercus spp. L.*, TADI = *Taxodium distichum (L.) Rich.*, TSME = *Tsuga mertensiana (Bong.) Carrière*, N/A = Not Available. The red background represents the SAT-sensitive tree-ring data, and the blue background represents the SPEI-sensitive tree-ring data.

Table S2. Climate observations, reanalyses, and model data used in this study.

Acronym (Reference)	Full name / URL	Time span	Temporal & spatial resolution	Variables used in this study
JRA-55 (*76*)	Japanese 55-year Reanalysis / jra.kishou.go.jp/JRA-55/index_en.html	1958-2022	Monthly, 6-hourly & 1.25° x 1.25°	All variables used in this study, except for SPEI6, SST, and PRCP
CRU TS (*100*)	Climatic Research Unit Time-Series Version 4.07 / crudata.uea.ac.uk/cru/data/hrg/	1901-2022	Monthly & 0.5° x 0.5°	SAT, PRCP
SPEIbase (*20*)	Global SPEI database Version 2.9 / spei.csic.es/	1901-2022	Monthly & 0.5° x 0.5°	SPEI6
HadISST (*101*)	Hadley Centre Sea Ice and Sea Surface Temperature / metoffice.gov.uk/hadobs/hadisst/	1870-2022	Monthly & 1.0° x 1.0°	SST
GPCP (*102*)	Global Precipitation Climatology Project / psl.noaa.gov/data/gridded/data.gpcp.html	1979-2022	Monthly & 2.5° x 2.5°	PRCP
CMIP6 (*71*)	Coupled Model Intercomparison Project Phase 6 / aims2.llnl.gov/search	1850-2100	Monthly, daily & Model-dependent	SAT, PRCP, SST, GPH250
ERA5 (*77*)	ECMWF Reanalysis v5 / cds.climate.copernicus.eu/	1940-2022	Monthly & 0.5° x 0.5°	GPH250
MERRA-2 (*78*)	Modern-Era Retrospective analysis for Research and Applications, version 2 / gmao.gsfc.nasa.gov/reanalysis/merra-2/	1980-2022	Monthly & 0.625° x 0.5°	GPH250
NCEP R1 (*79*)	NCEP/NCAR Reanalysis 1 / psl.noaa.gov/data/gridded/data.ncep.reanalysis.html	1948-2022	Monthly & 2.5° x 2.5°	GPH250
NCEP R2 (*80*)	NCEP-DOE Reanalysis 2 / psl.noaa.gov/data/gridded/data.ncep.reanalysis2.html	1979-2022	Monthly & 2.5° x 2.5°	GPH250
NOAA 20CR (*81*)	NOAA-CIRES-DOE 20th Century Reanalysis V3 / psl.noaa.gov/data/20thC_Rean/	1901-2022	Monthly & 1.0° x 1.0°	GPH250

Note: SAT = surface air temperature, SPEI6: 6 months standardized precipitation evapotranspiration index, SST: sea surface temperature, PRCP: precipitation, GPH250: geopotential height at 250 hPa.

Table S3. Information of tree-ring data used in the CGT index reconstruction.

No.	Name	Country	Lat. (°N)	Lon. (°E)	Elev. (m)	Species	Time span (CE)
Tree-ring data used in the CGT index reconstruction for both above-interannual and interannual timescale							
1	CHIN066	China	33.72	96.28	3950	JUTI	1374 - 2002
2	MONG034	Mongolia	49.41	107.57	1027	PISY	1730 - 2008
Tree-ring data used in the CGT index reconstruction for above-interannual timescale							
3	CA648	United States	40.53	-120.02	396	QUDG	1582 - 2004
4	CANA262	Canada	49.53	-91.22	450	PIST	1767 - 2004
5	CANA580	Canada	51.20	-114.30	1575	PSME	1575 - 2003
6	CHIN055	China	37.51	97.06	3780	JUPR	1237 - 2002
7	CHIN059	China	33.80	96.13	4060	JUTI	1480 - 2002
8	CHIN067	China	37.25	97.75	N/A	JUPR	2637 BC - 2011
9	CO614	United States	40.80	-105.18	1859	PIED	1508 - 2002
10	FL009	United States	28.30	-82.08	26	TADI	1622 - 2005
11	GERM152	Germany	51.17	8.95	420	FASY	1789 - 2005
12	GERM222	Germany	53.49	10.92	N/A	QURO	1766 - 2009
13	MT130	United States	46.82	-114.25	N/A	PSME	1307 - 2008
14	NJ007	United States	41.12	-74.47	366	QUPR	1577 - 2002
15	NORW030	Norway	69.92	23.11	73	PISY	1792 - 2015
16	RUS385	Russia	56.05	48.46	87	PISY	1802 - 2014
17	SWIT348	Switzerland	46.50	8.77	1400	PISY	1622 - 2002
18	WA139	United States	48.51	-118.75	965	PIPO	1600 - 2012
Tree-ring data used in the CGT index reconstruction for interannual timescale							
19	CANA491	Canada	51.24	-124.87	N/A	TSME	1673 - 2000
20	CANA528	Canada	49.31	-114.43	1838	LALY	1616 - 2010
21	CHIN072	China	33.73	112.23	1675	PITB	1774 - 2007
22	FINL047	Finland	68.92	28.48	N/A	PISY	1622 - 2001
23	FINL078	Finland	69.52	28.57	120	PISY	1700 - 2011
24	FINL082	Finland	69.26	27.40	200	PISY	1717 - 2011
25	KYRG014	Kyrgyzstan	42.41	78.96	3010	PCSH	1551 - 2005
26	MONG014	Mongolia	49.48	100.83	1800	LASI	1557 - 2002
27	MT119	United States	46.02	-113.37	2700	LALY	1026 - 2005
28	MT141	United States	48.97	-114.10	2260	LALY	1540 - 2004
29	MT142	United States	48.98	-114.25	1710	PSME	1499 - 2002
30	MT155	United States	48.38	-113.62	1890	PSME	1660 - 2001
31	MT157	United States	46.28	-113.15	2645	LALY	999 - 2013
32	OR098	United States	42.92	-122.05	2198	TSME	1510 - 2012
33	OR099	United States	42.97	-122.15	2221	TSME	1566 - 2012
34	OR101	United States	42.93	-122.02	2352	TSME	1508 - 2012
35	OR103	United States	42.91	-122.07	2198	TSME	1569 - 2012
36	PAKI036	Pakistan	36.15	74.18	N/A	PCSM	1387 - 2005
37	RUSS297	Russia	62.25	129.62	216	LAGM	1779 - 2013
38	WA148	United States	48.68	-121.32	1473	TSME	1746 - 2011

Note: Same as table S1.

Table S4. Nested reconstructions for the TEHD index.

No.	Nest Period	PCs with eigenvalues greater than 1.0	PCs used in the reconstruction	No. of used TRWs
Nests for above-interannual component reconstruction (1741-2000)				
1	1808-2000	PC1 to PC6	PC1, PC2, PC4	23
2	1806-1807	PC1 to PC6	PC1, PC4, PC6	22
3	1785-1805	PC1 to PC6	PC1, PC2, PC4, PC5	20
4	1782-1784	PC1 to PC6	PC1, PC4, PC5	19
5	1774-1781	PC1 to PC5	PC1, PC2, PC3	18
6	1741-1776	PC1, PC2, PC3	PC1	14
Nests for interannual component reconstruction (1674-2000)				
1	1850-2000	PC1 to PC6	PC1, PC2, PC5, PC6	18
2	1847-1849	PC1 to PC6	PC1, PC2, PC5, PC6	17
3	1842-1846	PC1 to PC5	PC1, PC2, PC3, PC5	15
4	1794-1841	PC1 to PC4	PC1, PC2, PC3, PC4	9
5	1715-1793	PC1, PC2, PC3	PC1, PC2	6
6	1674-1714	PC1, PC2	PC1, PC2	5

Table S5. Nested reconstructions for the CGT index.

No.	Nest Period	PCs with eigenvalues greater than 1.0	PCs used in the reconstruction	No. of used TRWs
Nests for above-interannual component reconstruction (1674-2000)				
1	1834-2000	PC1 to PC6	PC1, PC2, PC4, PC6	18
2	1722-1833	PC1, PC2, PC3	PC1, PC2	9
3	1674-1721	PC1, PC2	PC1, PC2	6
Nests for interannual component reconstruction (1708-2000)				
1	1838-2000	PC1 to PC8	PC1, PC2, PC3, PC4	22
2	1825-1837	PC1 to PC7	PC1, PC2, PC4, PC5	20
3	1759-1824	PC1 to PC6	PC1, PC2, PC4, PC6	15
4	1738-1758	PC1 to PC5	PC1, PC2, PC4	14
5	1723-1737	PC1 to PC4	PC1	13
6	1708-1722	PC1 to PC4	PC1	12

Table S6. List of CMIP6 climate models used in this study.

No.	Acronym	Institution / Country
1	ACCESS-CM2	Commonwealth Scientific and Industrial Research Organisation, Australian Research Council Centre of Excellence for Climate System Science / Australia
2	ACCESS-ESM1-5	Commonwealth Scientific and Industrial Research Organisation / Australia
3	AWI-CM-1-1-MR	Alfred Wegener Institute, Helmholtz Centre for Polar and Marine Research / Germany
4	CanESM5	Canadian Centre for Climate Modelling and Analysis, Environment and Climate Change Canada / Italy
5	CMCC-ESM2	Euro-Mediterranean Center on Climate Change
6<br>7<br>8	EC-Earth3<br>EC-Earth3-Veg<br>EC-Earth3-Veg-LR	EC-Earth consortium / Europe
9<br>10	FGOALS-f3-L<br>FGOALS-g3	Chinese Academy of Sciences / China
11	GFDL-ESM4	National Oceanic and Atmospheric Administration, Geophysical Fluid Dynamics Laboratory / USA
12<br>13	INM-CM4-8<br>INM-CM5-0	Institute for Numerical Mathematics, Russian Academy of Science / Russia
14	IPSL-CM6A-LR	Institute Pierre Simon Laplace / France
15	MIROC6	JAMSTEC (Japan Agency for Marine-Earth Science and Technology), AORI (Atmosphere and Ocean Research Institute, The University of Tokyo), NIES (National Institute for Environmental Studies), and R-CCS (RIKEN Center for Computational Science) / Japan
16<br>17	MPI-ESM1-2-HR<br>MPI-ESM1-2-LR	Max Planck Institute for Meteorology / Germany
18	MRI-ESM2-0	Meteorological Research Institute / Japan
19<br>20	NorESM2-LM<br>NorESM2-MM	NorESM Climate modeling Consortium consisting of CICERO (Center for International Climate and Environmental Research), MET-Norway (Norwegian Meteorological Institute), NERSC (Nansen Environmental and Remote Sensing Center), NILU (Norwegian Institute for Air Research), UiB (University of Bergen), UiO (University of Oslo) and UNI (Uni Research) / Norway
21	TaiESM1	Research Center for Environmental Changes, Academia Sinica / Taiwan

REFERENCES AND NOTES

1. S. Mukherjee, A. K. Mishra, Increase in compound drought and heatwaves in a warming world. *Geophys. Res. Lett.* **48**, e2020GL090617 (2021).

2. Z. Hao, F. Hao, Y. Xia, S. Feng, C. Sun, X. Zhang, Y. Fu, Y. Hao, Y. Zhang, Y. Meng, Compound droughts and hot extremes: Characteristics, drivers, changes, and impacts. *Earth Sci. Rev.* **235**, 104241 (2022).

3. D. Chen, S. Qiao, J. Yang, S. Tang, D. Zuo, G. Feng, Contribution of anthropogenic influence to the 2022-like Yangtze River valley compound heatwave and drought event. *NPJ Clim. Atmos. Sci.* **7**, 172 (2024).

4. C. Wang, Z. Li, Y. Chen, L. Ouyang, H. Zhao, J. Zhu, J. Wang, Y. Zhao, Characteristic changes in compound drought and heatwave events under climate change. *Atmos. Res.* **305**, 107440 (2024).

5. K. P. Tripathy, S. Mukherjee, A. K. Mishra, M. E. Mann, A. P. Williams, Climate change will accelerate the high-end risk of compound drought and heatwave events. *Proc. Natl. Acad. Sci. U.S.A.* **120**, e2219825120 (2023).

6. W. Li, B. Sun, H. Wang, B. Zhou, H. Li, R. Xue, M. Duan, X. Luo, W. Ai, Anthropogenic impact on the severity of compound extreme high temperature and drought/rain events in China. *NPJ Clim. Atmos. Sci.* **6**, 79 (2023).

7. A. P. Williams, E. R. Cook, J. E. Smerdon, B. I. Cook, J. T. Abatzoglou, K. Bolles, S. H. Baek, A. M. Badger, B. Livneh, Large contribution from anthropogenic warming to an emerging North American megadrought. *Science* **368**, 314–318 (2020).

8. N. N. Ridder, A. J. Pitman, S. Westra, A. Ukkola, H. X. Do, M. Bador, A. L. Hirsch, J. P. Evans, A. Di Luca, J. Zscheischler, Global hotspots for the occurrence of compound events. *Nat. Commun.* **11**, 5956 (2020).

9. P. Zhang, J.-H. Jeong, J.-H. Yoon, H. Kim, S. Y. S. Wang, H. W. Linderholm, K. Fang, X. Wu, D. Chen, Abrupt shift to hotter and drier climate over inner East Asia beyond the tipping point. *Science* **370**, 1095–1099 (2020).

10. C. Wang, Z. Li, Y. Chen, L. Ouyang, Y. Li, F. Sun, Y. Liu, J. Zhu, Drought-heatwave compound events are stronger in drylands. *Weather Clim. Extrem.* **42**, 100632 (2023).

11. X. Wang, Y. Li, Y. Chen, Y. Li, C. Wang, A. Kaldybayev, R. Gou, M. Luo, W. Duan, Intensification of heatwaves in Central Asia from 1981 to 2020 – Role of soil moisture reduction. *J. Hydrol.* **627**, 130395 (2023).

12. K. C. Bolles, A. P. Williams, E. R. Cook, B. I. Cook, D. A. Bishop, Tree-ring reconstruction of the atmospheric ridging feature that causes flash drought in the Central United States since 1500. *Geophys. Res. Lett.* **48**, e2020GL091271 (2021).

13. Y.-W. Seo, K.-J. Ha, Changes in land-atmosphere coupling increase compound drought and heatwaves over northern East Asia. *NPJ Clim. Atmos. Sci.* **5**, 100 (2022).

14. D. L. Schumacher, J. Keune, C. C. van Heerwaarden, J. Vilà-Guerau de Arellano, A. J. Teuling, D. G. Miralles, Amplification of mega-heatwaves through heat torrents fuelled by upwind drought. *Nat. Geosci.* **12**, 712–717 (2019).

15. F. Cai, C. Liu, D. Gerten, S. Yang, T. Zhang, S. Lin, J. Kurths, Pronounced spatial disparity of projected heatwave changes linked to heat domes and land-atmosphere coupling. *NPJ Clim. Atmos. Sci.* **7**, 225 (2024).

16. E. Rousi, K. Kornhuber, G. Beobide-Arsuaga, F. Luo, D. Coumou, Accelerated western European heatwave trends linked to more-persistent double jets over Eurasia. *Nat. Commun.* **13**, 3851 (2022).

17. D. G. Miralles, A. J. Teuling, C. C. van Heerwaarden, J. Vilà-Guerau de Arellano, Mega-heatwave temperatures due to combined soil desiccation and atmospheric heat accumulation. *Nat. Geosci.* **7**, 345–349 (2014).

18. F. Ji, Z. Wu, J. Huang, E. P. Chassignet, Evolution of land surface air temperature trend. *Nat. Clim. Change* **4**, 462–466 (2014).

19. U. Büntgen, K. Allen, K. J. Anchukaitis, D. Arseneault, É. Boucher, A. Bräuning, S. Chatterjee, P. Cherubini, O. V. Churakova, C. Corona, F. Gennaretti, J. Grießinger, S. Guillet, J. Guiot, B. Gunnarson, S. Helama, P. Hochreuther, M. K. Hughes, P. Huybers, A. V. Kirdyanov, P. J. Krusic, J. Ludescher, W. J.-H. Meier, V. S. Myglan, K. Nicolussi, C. Oppenheimer, F. Reinig, M. W. Salzer, K. Seftigen, A. R. Stine, M. Stoffel, S. St. George, E. Tejedor, A. Trevino, V. Trouet, J. Wang, R. Wilson, B. Yang, G. Xu, J. Esper, The influence of decision-making in tree ring-based climate reconstructions. *Nat. Commun.* **12**, 3411 (2021).

20. S. M. Vicente-Serrano, S. Beguería, J. I. López-Moreno, A multiscalar drought index sensitive to global warming: The standardized precipitation evapotranspiration index. *J. Clim.* **23**, 1696–1718 (2010).

21. L. M. Rasmijn, G. van der Schrier, R. Bintanja, J. Barkmeijer, A. Sterl, W. Hazeleger, Future equivalent of 2010 Russian heatwave intensified by weakening soil moisture constraints. *Nat. Clim. Change* **8**, 381–385 (2018).

22. E. Seo, M.-I. Lee, S. D. Schubert, R. D. Koster, H.-S. Kang, Investigation of the 2016 Eurasia heat wave as an event of the recent warming. *Environ. Res. Lett.* **15**, 114018 (2020).

23. D. G. Miralles, M. J. van den Berg, A. J. Teuling, R. A. M. de Jeu, Soil moisture-temperature coupling: A multiscale observational analysis. *Geophys. Res. Lett.* **39**, L21707 (2012).

24. K. Deng, S. Yang, M. Ting, A. Lin, Z. Wang, An intensified mode of variability modulating the summer heat waves in Eastern Europe and Northern China. *Geophys. Res. Lett.* **45**, 11,361–11,369 (2018).

25. M.-H. Lee, S. Lee, H.-J. Song, C.-H. Ho, The recent increase in the occurrence of a boreal summer teleconnection and its relationship with temperature extremes. *J. Clim.* **30**, 7493–7504 (2017).

26. C. D. W. Rogers, K. Kornhuber, S. E. Perkins-Kirkpatrick, P. C. Loikith, D. Singh, Sixfold increase in historical Northern Hemisphere concurrent large heatwaves driven by warming and changing atmospheric circulations. *J. Clim.* **35**, 1063–1078 (2022).

27. S.-K. Min, Y.-H. Kim, S.-M. Lee, S. Sparrow, S. Li, F. C. Lott, P. A. Stott, Quantifying human impact on the 2018 summer longest heat wave in South Korea. *Bull. Am. Meteorol. Soc.* **101**, S103–S108 (2020).

28. P. Yiou, J. Cattiaux, D. Faranda, N. Kadygrov, A. Jézéquel, P. Naveau, A. Ribes, Y. Robin, S. Thao, G. J. van Oldenborgh, M. Vrac, Analyses of the Northern European summer heatwave of 2018. *Bull. Am. Meteorol. Soc.* **101**, S35–S40 (2020).

29. C. Zhou, D. Chen, K. Wang, A. Dai, D. Qi, Conditional attribution of the 2018 summer extreme heat over Northeast China: Roles of urbanization, global warming, and warming-induced circulation changes. *Bull. Am. Meteorol. Soc.* **101**, S71–S76 (2020).

30. J. Zhang, H. Chen, X. Fang, Z. Yin, R. Hu, Warming-induced hydrothermal anomaly over the Earth's three Poles amplifies concurrent extremes in 2022. *NPJ Clim. Atmos. Sci.* **7**, 8 (2024).

31. K. Kornhuber, S. Osprey, D. Coumou, S. Petri, V. Petoukhov, S. Rahmstorf, L. Gray, Extreme weather events in early summer 2018 connected by a recurrent hemispheric wave-7 pattern. *Environ. Res. Lett.* **14**, 054002 (2019).

32. T. Zhang, Y. Deng, J. Chen, S. Yang, Y. Dai, An energetics tale of the 2022 mega-heatwave over central-eastern China. *NPJ Clim. Atmos. Sci.* **6**, 162 (2023).

33. D. Barriopedro, E. M. Fischer, J. Luterbacher, R. M. Trigo, R. García-Herrera, The hot summer of 2010: Redrawing the temperature record map of Europe. *Science* **332**, 220–224 (2011).

34. J. I. Christian, J. B. Basara, E. D. Hunt, J. A. Otkin, X. Xiao, Flash drought development and cascading impacts associated with the 2010 Russian heatwave. *Environ. Res. Lett.* **15**, 094078 (2020).

35. S. Russo, J. Sillmann, E. M. Fischer, Top ten European heatwaves since 1950 and their occurrence in the coming decades. *Environ. Res. Lett.* **10**, 124003 (2015).

36. D. Shaposhnikov, B. Revich, T. Bellander, G. B. Bedada, M. Bottai, T. Kharkova, E. Kvasha, E. Lezina, T. Lind, E. Semutnikova, G. Pershagen, Mortality related to air pollution with the Moscow heat wave and wildfire of 2010. *Epidemiology* **25**, 359–364 (2014).

37. Y. Wang, X. Yuan, High temperature accelerates onset speed of the 2022 unprecedented flash drought over the Yangtze River Basin. *Geophys. Res. Lett.* **50**, e2023GL105375 (2023).

38. A. Seim, J. A. Schultz, C. Beck, A. Bräuning, P. J. Krusic, C. Leland, O. Byambasuren, E. Liang, X. Wang, J.-H. Jeong, H. W. Linderholm, Evaluation of tree growth relevant atmospheric circulation patterns for geopotential height field reconstructions for Asia. *J. Clim.* **31**, 4391–4401 (2018).

39. A. Seim, J. A. Schultz, C. Leland, N. Davi, O. Byambasuren, E. Liang, X. Wang, C. Beck, H. W. Linderholm, N. Pederson, Synoptic-scale circulation patterns during summer derived from tree rings in mid-latitude Asia. *Clim. Dyn.* **49**, 1917–1931 (2017).

40. H. Lopez, S.-K. Lee, S. Dong, G. Goni, B. Kirtman, R. Atlas, A. Kumar, East Asian monsoon as a modulator of U.S. great plains heat waves. *J. Geophys. Res.* **124**, 6342–6358 (2019).

41. D. Hodges, Z. Pu, Characteristics and variations of low-level jets and environmental factors associated with summer precipitation extremes over the great plains. *J. Clim.* **32**, 5123–5144 (2019).

42. T. Cowan, G. C. Hegerl, I. Colfescu, M. Bollasina, A. Purich, G. Boschat, Factors contributing to record-breaking heat waves over the great plains during the 1930s dust bowl. *J. Clim.* **30**, 2437–2461 (2017).

43. R. D'Arrigo, G. Jacoby, R. Wilson, F. Panagiotopoulos, A reconstructed Siberian High index since A.D. 1599 from Eurasian and North American tree rings. *Geophys. Res. Lett.* **32**, L05705 (2005).

44. R. D. D'Arrigo, E. R. Cook, M. E. Mann, G. C. Jacoby, Tree-ring reconstructions of temperature and sea-level pressure variability associated with the warm-season Arctic Oscillation since AD 1650. *Geophys. Res. Lett.* **30**, 1549 (2003).

45. C. K. Folland, J. Knight, H. W. Linderholm, D. Fereday, S. Ineson, J. W. Hurrell, The summer North Atlantic oscillation: Past, present, and future. *J. Clim.* **22**, 1082–1103 (2009).

46. V. Trouet, F. Babst, M. Meko, Recent enhanced high-summer North Atlantic Jet variability emerges from three-century context. *Nat. Commun.* **9**, 180 (2018).

47. V. Trouet, A. H. Taylor, Multi-century variability in the Pacific North American circulation pattern reconstructed from tree rings. *Clim. Dyn.* **35**, 953–963 (2010).

48. G. Xu, E. Broadman, I. Dorado-Liñán, L. Klippel, M. Meko, U. Büntgen, T. De Mil, J. Esper, B. Gunnarson, C. Hartl, P. J. Krusic, H. W. Linderholm, F. C. Ljungqvist, F. Ludlow, M. Panayotov, A. Seim, R. Wilson, D. Zamora-Reyes, V. Trouet, Jet stream controls on European climate and agriculture since 1300 CE. *Nature* **634**, 600–608 (2024).

49. Y. Liu, K. M. Cobb, H. Song, Q. Li, C.-Y. Li, T. Nakatsuka, Z. An, W. Zhou, Q. Cai, J. Li, S. W. Leavitt, C. Sun, R. Mei, C.-C. Shen, M.-H. Chan, J. Sun, L. Yan, Y. Lei, Y. Ma, X. Li, D. Chen, H. W. Linderholm, Recent enhancement of central Pacific El Niño variability relative to last eight centuries. *Nat. Commun.* **8**, 15386 (2017).

50. W. E. Wright, B. T. Guan, Y.-H. Tseng, E. R. Cook, K. Y. Wei, S. T. Chang, Reconstruction of the springtime East Asian Subtropical Jet and Western Pacific pattern from a millennial-length Taiwanese tree-ring chronology. *Clim. Dyn.* **44**, 1645–1659 (2015).

51. S. T. Gray, L. J. Graumlich, J. L. Betancourt, G. T. Pederson, A tree-ring based reconstruction of the Atlantic Multidecadal Oscillation since 1567 A.D. *Geophys. Res. Lett.* **31**, L12205 (2004).

52. R. D'Arrigo, R. Villalba, G. Wiles, Tree-ring estimates of Pacific decadal climate variability. *Clim. Dyn.* **18**, 219–224 (2001).

53. Q. Ding, B. Wang, Circumglobal teleconnection in the Northern Hemisphere summer. *J. Clim.* **18**, 3483–3505 (2005).

54. Q. Ding, B. Wang, J. M. Wallace, G. Branstator, Tropical–extratropical teleconnections in boreal summer: Observed interannual variability. *J. Clim.* **24**, 1878–1896 (2011).

55. H. Wang, B. Wang, F. Huang, Q. Ding, J.-Y. Lee, Interdecadal change of the boreal summer circumglobal teleconnection (1958–2010). *Geophys. Res. Lett.* **39**, L12704 (2012).

56. J. Kysely, J. Kim, Mortality during heat waves in South Korea, 1991 to 2005: How exceptional was the 1994 heat wave? *Clim. Res.* **38**, 105–116 (2009).

57. G. C. Satyanarayana, D. V. B. Rao, Phenology of heat waves over India. *Atmos. Res.* **245**, 105078 (2020).

58. N. Khan, S. Shahid, T. Ismail, K. Ahmed, N. Nawaz, Trends in heat wave related indices in Pakistan. *Stoch. Environ. Res. Risk Assess.* **33**, 287–302 (2019).

59. J. Kyselý, Mortality and displaced mortality during heat waves in the Czech Republic. *Int. J. Biometeorol.* **49**, 91–97 (2004).

60. D. Graczyk, Z. W. Kundzewicz, A. Choryński, E. J. Førland, I. Pińskwar, M. Szwed, Heat-related mortality during hot summers in Polish cities. *Theor. Appl. Climatol.* **136**, 1259–1273 (2019).

61. X. Sun, Q. Ding, S.-Y. S. Wang, D. Topál, Q. Li, C. Castro, H. Teng, R. Luo, Y. Ding, Enhanced jet stream waviness induced by suppressed tropical Pacific convection during boreal summer. *Nat. Commun.* **13**, 1288 (2022).

62. M. Gao, J. Yang, D. Gong, P. Shi, Z. Han, S.-J. Kim, Footprints of Atlantic multidecadal oscillation in the low-frequency variation of extreme high temperature in the Northern Hemisphere. *J. Clim.* **32**, 791–802 (2019).

63. H. Teng, R. Leung, G. Branstator, J. Lu, Q. Ding, Warming pattern over the Northern Hemisphere midlatitudes in boreal summer 1979–2020. *J. Clim.* **35**, 3479–3494 (2022).

64. J. Wang, B. Yang, F. C. Ljungqvist, J. Luterbacher, T. J. Osborn, K. R. Briffa, E. Zorita, Internal and external forcing of multidecadal Atlantic climate variability over the past 1,200 years. *Nat. Geosci.* **10**, 512–517 (2017).

65. C. He, A. C. Clement, S. M. Kramer, M. A. Cane, J. M. Klavans, T. M. Fenske, L. N. Murphy, Tropical Atlantic multidecadal variability is dominated by external forcing. *Nature* **622**, 521–527 (2023).

66. D. Zanchettin, A. Rubino, Accelerated North Atlantic surface warming reshapes the Atlantic Multidecadal Variability. *Commun. Earth Environ.* **5**, 639 (2024).

67. S.-Y. Wang, R. R. Gillies, Observed change in Sahel rainfall, circulations, African Easterly waves, and Atlantic hurricanes Since 1979. *Intl. J. Geophys.* **2011**, 259529 (2011).

68. B. Dong, R. Sutton, Dominant role of greenhouse-gas forcing in the recovery of Sahel rainfall. *Nat. Clim. Change* **5**, 757–760 (2015).

69. T. Nakanishi, Y. Tachibana, Y. Ando, Possible semi-circumglobal teleconnection across Eurasia driven by deep convection over the Sahel. *Clim. Dyn.* **57**, 2287–2299 (2021).

70. D. W. J. Thompson, J. M. Wallace, Regional climate impacts of the Northern Hemisphere annular mode. *Science* **293**, 85–89 (2001).

71. V. Eyring, S. Bony, G. A. Meehl, C. A. Senior, B. Stevens, R. J. Stouffer, K. E. Taylor, Overview of the Coupled Model Intercomparison Project Phase 6 (CMIP6) experimental design and organization. *Geosci. Model Dev.* **9**, 1937–1958 (2016).

72. N. P. Gillett, H. Shiogama, B. Funke, G. Hegerl, R. Knutti, K. Matthes, B. D. Santer, D. Stone, C. Tebaldi, The Detection and Attribution Model Intercomparison Project (DAMIP v1.0) contribution to CMIP6. *Geosci. Model Dev.* **9**, 3685–3697 (2016).

73. B. van den Hurk, H. Kim, G. Krinner, S. I. Seneviratne, C. Derksen, T. Oki, H. Douville, J. Colin, A. Ducharne, F. Cheruy, N. Viovy, M. J. Puma, Y. Wada, W. Li, B. Jia, A. Alessandri, D. M. Lawrence, G. P. Weedon, R. Ellis, S. Hagemann, J. Mao, M. G. Flanner, M. Zampieri, S. Materia, R. M. Law, J. Sheffield, LS3MIP (v1.0) contribution to CMIP6: The Land Surface, Snow and Soil moisture Model Intercomparison Project—Aims, setup and expected outcome. *Geosci. Model Dev.* **9**, 2809–2832 (2016).

74. C. Deser, F. Lehner, K. B. Rodgers, T. Ault, T. L. Delworth, P. N. DiNezio, A. Fiore, C. Frankignoul, J. C. Fyfe, D. E. Horton, J. E. Kay, R. Knutti, N. S. Lovenduski, J. Marotzke, K. A. McKinnon, S. Minobe, J. Randerson, J. A. Screen, I. R. Simpson, M. Ting, Insights from Earth system model initial-condition large ensembles and future prospects. *Nat. Clim. Change* **10**, 277–286 (2020).

75. S. E. Perkins-Kirkpatrick, S. C. Lewis, Increasing trends in regional heatwaves. *Nat. Commun.* **11**, 3357 (2020).

76. S. Kobayashi, Y. Ota, Y. Harada, A. Ebita, M. Moriya, H. Onoda, K. Onogi, H. Kamahori, C. Kobayashi, H. Endo, K. Miyaoka, K. Takahashi, The JRA-55 reanalysis: General specifications and basic characteristics. *J Meteorol Soc Jpn.* **93**, 5–48 (2015).

77. H. Hersbach, B. Bell, P. Berrisford, S. Hirahara, A. Horányi, J. Muñoz-Sabater, J. Nicolas, C. Peubey, R. Radu, D. Schepers, A. Simmons, C. Soci, S. Abdalla, X. Abellan, G. Balsamo, P. Bechtold, G. Biavati, J. Bidlot, M. Bonavita, G. De Chiara, P. Dahlgren, D. Dee, M. Diamantakis, R. Dragani, J. Flemming, R. Forbes, M. Fuentes, A. Geer, L. Haimberger, S. Healy, R. J. Hogan, E. Hólm, M. Janisková, S. Keeley, P. Laloyaux, P. Lopez, C. Lupu, G. Radnoti, P. de Rosnay, I. Rozum, F. Vamborg, S. Villaume, J.-N. Thépaut, The ERA5 global reanalysis. *Q. J. Roy. Meteorol. Soc.* **146**, 1999–2049 (2020).

78. R. Gelaro, W. McCarty, M. J. Suárez, R. Todling, A. Molod, L. Takacs, C. A. Randles, A. Darmenov, M. G. Bosilovich, R. Reichle, K. Wargan, L. Coy, R. Cullather, C. Draper, S. Akella, V. Buchard, A. Conaty, A. M. da Silva, W. Gu, G.-K. Kim, R. Koster, R. Lucchesi, D. Merkova, J. E. Nielsen, G. Partyka, S. Pawson, W. Putman, M. Rienecker, S. D. Schubert, M. Sienkiewicz,

B. Zhao, The Modern-Era Retrospective Analysis for Research and Applications, Version 2 (MERRA-2). *J. Clim.* **30**, 5419–5454 (2017).

79. E. Kalnay, M. Kanamitsu, R. Kistler, W. Collins, D. Deaven, L. Gandin, M. Iredell, S. Saha, G. White, J. Woollen, Y. Zhu, M. Chelliah, W. Ebisuzaki, W. Higgins, J. Janowiak, K. C. Mo, C. Ropelewski, J. Wang, A. Leetmaa, R. Reynolds, R. Jenne, D. Joseph, The NCEP/NCAR 40-year reanalysis project. *Bull. Am. Meteorol. Soc.* **77**, 437–472 (1996).

80. M. Kanamitsu, W. Ebisuzaki, J. Woollen, S.-K. Yang, J. J. Hnilo, M. Fiorino, G. L. Potter, NCEP–DOE AMIP-II Reanalysis (R-2). *Bull. Am. Meteorol. Soc.* **83**, 1631–1644 (2002).

81. L. C. Slivinski, G. P. Compo, J. S. Whitaker, P. D. Sardeshmukh, B. S. Giese, C. McColl, R. Allan, X. Yin, R. Vose, H. Titchner, J. Kennedy, L. J. Spencer, L. Ashcroft, S. Brönnimann, M. Brunet, D. Camuffo, R. Cornes, T. A. Cram, R. Crouthamel, F. Domínguez-Castro, J. E. Freeman, J. Gergis, E. Hawkins, P. D. Jones, S. Jourdain, A. Kaplan, H. Kubota, F. L. Blancq, T.-C. Lee, A. Lorrey, J. Luterbacher, M. Maugeri, C. J. Mock, G. W. K. Moore, R. Przybylak, C. Pudmenzky, C. Reason, V. C. Slonosky, C. A. Smith, B. Tinz, B. Trewin, M. A. Valente, X. L. Wang, C. Wilkinson, K. Wood, P. Wyszyński, Towards a more reliable historical reanalysis: Improvements for version 3 of the Twentieth Century Reanalysis system. *Q. J. Roy. Meteorol. Soc.* **145**, 2876–2908 (2019).

82. Y. Benjamini, Y. Hochberg, Controlling the false discovery rate: A practical and powerful approach to multiple testing. *J. R. Stat. Soc. B. Methodol.* **57**, 289–300 (1995).

83. D. S. Wilks, "The stippling shows statistically significant grid points": How research results are routinely overstated and overinterpreted, and what to do about it. *Bull. Am. Meteorol. Soc.* **97**, 2263–2273 (2016).

84. D. S. Wilks, On "field significance" and the false discovery rate. *J. Appl. Meteorol. Climatol.* **45**, 1181–1189 (2006).

85. T. M. Melvin, K. R. Briffa, CRUST: Software for the implementation of Regional Chronology Standardisation: Part 1. Signal-Free RCS. *Dendrochronologia* **32**, 7–20 (2014).

86. T. M. Melvin, K. R. Briffa, CRUST: Software for the implementation of Regional Chronology Standardisation: Part 2. Further RCS options and recommendations. *Dendrochronologia* **32**, 343–356 (2014).

87. T. M. L. Wigley, K. R. Briffa, P. D. Jones, On the average value of correlated time series, with applications in dendroclimatology and hydrometeorology. *J. Appl. Meteorol. Climatol.* **23**, 201–213 (1984).

88. F. Shi, B. Yang, L. von Gunten, C. Qin, Z. Wang, Ensemble empirical mode decomposition for tree-ring climate reconstructions. *Theor. Appl. Climatol.* **109**, 233–243 (2012).

89. Z. H. Wu, N. E. Huang, Ensemble empirical mode decomposition: A noise-assisted data analysis method. *Adv. Adapt. Data Anal.* **01**, 1–41 (2009).

90. H. F. Kaiser, The application of electronic computers to factor analysis. *Educ. Psychol. Meas.* **20**, 141–151 (1960).

91. P. Peduzzi, J. Concato, E. Kemper, T. R. Holford, A. R. Feinstein, A simulation study of the number of events per variable in logistic regression analysis. *J. Clin. Epidemiol.* **49**, 1373–1379 (1996).

92. E. R. Cook, D. M. Meko, D. W. Stahle, M. K. Cleaveland, Drought reconstructions for the continental United States. *J. Clim.* **12**, 1145–1162 (1999).

93. K. Takaya, H. Nakamura, A formulation of a phase-independent wave-activity flux for stationary and migratory quasigeostrophic eddies on a zonally varying basic flow. *J. Atmos. Sci.* **58**, 608–627 (2001).

94. C. H. B. Priestley, R. J. Taylor, On the assessment of surface heat flux and evaporation using large-scale parameters. *Mon. Weather Rev.* **100**, 81–92 (1972).

95. R. G. Allen, L. S. Pereira, D. Raes, M. Smith, Crop evapotranspiration-Guidelines for computing crop water requirements-FAO Irrigation and drainage paper 56. Fao, Rome 300, D05109 (1998).

96. C. W. Thornthwaite, An approach toward a rational classification of climate. *Geogr. Rev.* **38**, 55–94 (1948).

97. K. E. Trenberth, D. J. Shea, Atlantic hurricanes and natural variability in 2005. *Geophys. Res. Lett.* **33**, L12704 (2006).

98. B. J. Henley, J. Gergis, D. J. Karoly, S. Power, J. Kennedy, C. K. Folland, A tripole index for the interdecadal Pacific oscillation. *Clim. Dyn.* **45**, 3077–3090 (2015).

99. C. He, X. Chen, M. Collins, F. Song, Y. Hu, X. Jiang, Y. Liu, Y. Ding, W. Zhou, Rising geopotential height under global warming. *Clim. Dyn.* **62**, 5769–5786 (2024).

100. I. Harris, T. J. Osborn, P. Jones, D. Lister, Version 4 of the CRU TS monthly high-resolution gridded multivariate climate dataset. *Sci. Data* **7**, 109 (2020).

101. N. A. Rayner, D. E. Parker, E. B. Horton, C. K. Folland, L. V. Alexander, D. P. Rowell, E. C. Kent, A. Kaplan, Global analyses of sea surface temperature, sea ice, and night marine air temperature since the late nineteenth century. *J. Geophys. Res. Atmos.* **108**, 4407 (2003).

102. R. F. Adler, G. J. Huffman, A. Chang, R. Ferraro, P.-P. Xie, J. Janowiak, B. Rudolf, U. Schneider, S. Curtis, D. Bolvin, A. Gruber, J. Susskind, P. Arkin, E. Nelkin, The Version-2 Global Precipitation Climatology Project (GPCP) monthly precipitation analysis (1979–present). *J. Hydrometeorol.* **4**, 1147–1167 (2003).
